# Characterization of γδ T Cells from Zebrafish Provides Insights into Their Important Role in Adaptive Humoral Immunity

**DOI:** 10.3389/fimmu.2016.00675

**Published:** 2017-01-09

**Authors:** Feng Wan, Chong-bin Hu, Jun-xia Ma, Ke Gao, Li-xin Xiang, Jian-zhong Shao

**Affiliations:** ^1^College of Life Sciences, Zhejiang University, Hangzhou, China; ^2^Key Laboratory for Cell and Gene Engineering of Zhejiang Province, Hangzhou, China; ^3^Laboratory for Marine Biology and Biotechnology, Qingdao National Laboratory for Marine Science and Technology, Qingdao, China

**Keywords:** zebrafish γδ T cells, identification, origin of T cell subset, antigen-presenting cells, adaptive humoral immunity, mucosal IgZ antibody

## Abstract

γδ T cells represent an evolutionarily primitive T cell subset characterized by distinct T cell receptors (TCRs) and innate and adaptive immune functions. However, the presence of this T cell subset in ancient vertebrates remains unclear. In this study, γδ T cells from a zebrafish (*Danio rerio*) model were subjected to molecular and cellular characterizations. The constant regions of zebrafish TCR-γ (*Dr*TRGC) and δ (*Dr*TRDC) were initially identified. Zebrafish γδ T cells accounted for 7.7–20.5% of the total lymphocytes in spleen, head kidney, peripheral blood, skin, gill, and intestine tissues. They possess typical morphological features of lymphocytes with a surface phenotype of γ^+^δ^+^CD4^−^CD8^+^. Zebrafish γδ T cells functionally showed a potent phagocytic ability to both soluble and particulate antigens. They can also act as an antigen-presenting cell to initiate antigen (KLH)-specific CD4^+^ T_KLH_ cell activation and to induce B cell proliferation and IgM production. Particularly, zebrafish γδ T cells also play a critical role in antigen-specific IgZ production in intestinal mucus. These findings demonstrated that γδ T cells had been originated as early as teleost fish, which providing valuable insights into the evolutionary history of T cell subset. It is anticipated that this study would be used as a guide to develop a zebrafish model for the cross-species investigation of γδ T cell biology.

## Introduction

T cells are fundamental players of adaptive immune responses in vertebrates. The T cell receptor (TCR) is expressed as a heterodimeric protein on the cell surface of all T cells and confers the exquisite specificity to foreign antigens that typifies the adaptive immune system. T cells can be divided into two subsets on the basis of expressed TCR genes: αβ T cells and γδ T cells. γδ T cells are a distinct subset of CD3^+^ T cells containing TCRs encoded by Vγ and Vδ gene segments, which were first shown to exist in humans after the genes encoding their TCRs were cloned in the mid-1980s (1, 2). The discovery of γδ T cells is based on the structural similarities between their TCRs and those of αβ T cells. In contrast to conventional αβ T cells, γδ T cell repertoire is rather restricted because canonical γδ TCRs are expressed in specific anatomical locations (3). γδ T cells possess unique features; for instance, they do not display MHC restriction, and they do not recognize peptides processed from complex protein antigens by antigen-presenting cells (APCs); instead, γδ T cells distinguish unconventional antigens, such as phosphorylated microbial metabolites and lipid antigens (4). Moreover, it is not needed the presentation of those antigens by MHC-I and MHC-II, which is in agreement with the absence of CD4 or CD8 expression in the majority of γδ T cells (5). In human peripheral blood, γδ T cells constitute 2–10% of the total T cell pools. High proportions of γδ T cells are also found in the intestines, lungs, reproductive tract, and skin; as such, these cells may play a crucial role in mucosal immunity (6). γδ T cells contain TCRs that also function as pattern recognition receptors; for these reasons, these cells have been considered an evolutionarily primitive lymphocyte population functionally characterized “in between” innate and adaptive immune systems (7). Therefore, γδ T cells possibly link innate immunity to adaptive immunity, and thus they have become much attractive for the study of regulatory mechanisms across innate and adaptive immunity. On the other hand, γδ T cells may also be used as a model system to elucidate lymphocyte evolution, which is a key event in adaptive immunity originating from ancient vertebrates to humans (8). To date, γδ T cells have been extensively identified in humans and other mammals, including mice, sheep, cattle, and pigs; nevertheless, their existence or occurrence in ancient vertebrates remains poorly understood (9). Therefore, γδ T cells should be identified in evolutionarily lower vertebrates.

In evolutionary terms, teleost fish possess a complex innate immunity; they also comprise an ancient recognizable adaptive immune system containing T and B lymphocytes with somatically rearranged antigen receptors, MHC molecules, oldest immunoglobulins (Igs) in systematic (IgM) and mucosal (IgZ/T) immune systems, and immunological memory (10). Thus, teleost fish have been used as an efficient model organism to determine fundamental immunological events, such as the cooperation between innate immunity and adaptive immunity during the early evolution of adaptive immune systems and the origin of lymphocytes with typical adaptive natures from innate immune cells. Using this model, we may gain insights into the functional development of adaptive immune systems throughout vertebrate evolution. As a primitive lymphocyte subset that possibly originated in early evolutionary stages, γδ T cells may play prominent functions in teleost fish. Hence, γδ T cells in fish should be investigated to obtain key information regarding the ontogeny of innate and adaptive immunity systems in vertebrates.

In our study, γδ T cells from a zebrafish (*Danio rerio*) model were subjected to molecular and cellular identification and functional characterization. We found that zebrafish γδ T cells share typical morphological features of lymphocytes with a surface phenotype of γ^+^δ^+^CD4^−^CD8^+^ and potently phagocytose soluble and particulate antigens. *In vitro* antigen presentation and *in vivo* antibody blockade assays revealed that zebrafish γδ T cells can initiate Ag-specific CD4^+^ T cell proliferation and subsequently induce B cell activation and IgM production. This finding suggested that γδ T cells participate in the full activation of the systematic adaptive humoral immunity in zebrafish. Zebrafish γδ T cells also play a critical role in antigen-specific mucosal IgZ production in the intestinal mucus. These observations showed the innate-like nature of teleost γδ T cells in the interface of innate and adaptive immunities. These cells also comprise a major population of APCs functioning in systematic and mucosal adaptive immunities. To our knowledge, this study is the first to demonstrate the existence of γδ T cells that functionally link innate and adaptive immunities in a fish species. This study not only provided further insights into fish immunology but also helped enhance our cross-species understanding of the evolutionary history of the γδ T family and its cellular regulatory networks. Therefore, zebrafish may be used as a new model organism to investigate γδ T cell biology and γδ T cell-mediated diseases because of the molecular and functional conservation of γδ T cells between teleost fish and mammals and the crucial roles of γδ T cells in immune regulation, infectious diseases, and autoimmune disorders.

## Materials and Methods

### Experimental Fish

Wild-type AB zebrafish were bred and maintained in a circulating water bath at 28°C under standard conditions (11). All fish used in the experiments were offspring of a single AB strain parent pair after five generations of partial inbreeding (12). Healthy fish, as determined by their general appearance and activity level, was used in our study. All animal work in this paper was conducted according to relevant national and international guidelines. All animal care and experimental procedures were approved by the Committee on Animal Care and Use and the Committee on the Ethic of Animal Experiments of Zhejiang University.

### Molecular Cloning

The constant regions of the cDNAs of zebrafish TCR-α, TCR-β, TCR-γ, and TCR-δ (*Dr*TRAC, *Dr*TRBC, *Dr*TRGC, and *Dr*TRDC, respectively) were cloned from spleen and intestine tissues. Total RNAs were extracted using RNAiso Plus (TaKaRa). cDNA sequences were obtained through RT-PCR by using primers designed in accordance with the predicted sequences in NCBI (AF425590.1, AY973884.1, and CAK05280.1; Table S1 in Supplementary Material) (13–16). Gene organizations (intron/exon boundaries) were elucidated by comparing the cDNAs with genome sequences, and figures were drawn using GeneMaper 2.5. Protein sequence alignment was generated using Clustal X (1.81), and phylogenetic analyses were conducted in MEGA version 6.0.

### Preparation of Recombinant Proteins

For recombinant protein preparation, cDNAs encoding the extracellular region of *Dr*TRAC, *Dr*TRBC, *Dr*TRGC, and *Dr*TRDC were amplified through RT-PCR by using primers containing an *Eco*RI site added to the 5′ end and a *Xho*I site added to the 3′ end. The PCR products were digested and ligated into pET28a (Invitrogen Life Technologies). Then, plasmid DNAs were transformed into *Escherichia coli* Rosetta (Novagen). Single colony was inoculated into 100 mL of Luria–Bertani medium containing kanamycin (50 µg/mL) and shaken at 37°C until OD_600_ reached 0.6. Afterward, isopropyl-β-d-thiogalactoside was added to a final concentration of 1 mM. The culture was shaken continually at 37°C for 6 h. Protein expression levels were assessed through 10% SDS-PAGE followed by Coomassie brilliant blue R250 staining.

### Prediction of Abs

Abs against *Dr*TRGC, *Dr*TRDC, *Dr*TRAC, *Dr*TRBC, and CD8α were produced by an approach based on the prediction of antigen epitopes on the surfaces of these molecules (17). Briefly, ABCPred, BepiPred, MAPPP, and IEDB online software were used to search the epitope sequences (Table S2 in Supplementary Material), and their hydrophilic and antigen indices were evaluated by using DNAStar (18). Their 3D structures were predicted by utilizing SWISS-MODEL to detect whether the predicted antigen epitopes were exposed on the surfaces of the corresponding proteins. The specificity of the predicted sequences was confirmed by BLAST. The amino acid sequences of the antigen epitopes were chemically synthesized, purified through HPLC, and coupled to OVA at a ratio of 10:10 mg (carrier/peptide). New Zealand white rabbits (~1.5 kg) and Balb/c mice (~30 g) were immunized with OVA-coupled peptides (1 mg for rabbits and 0.1 mg for mice, respectively) in CFA on days 1 and 3 and in IFA on days 28 and 35. A week after the final immunization was administered, the animals were bled when Ab titers were above 1:10,000, as determined by ELISA with recombinant proteins adsorbed onto a solid phase. Abs were affinity purified by protein-G agarose as previously described (19). The specificity of Abs was detected through Western blot analyses, in which the blocking peptides (5 µg/mL) were also included (19, 20). Other Abs, including anti-*Dr*CD4, anti-*Dr*CD80/86, anti-*Dr*CD83, anti-*Dr*CD154, anti-*Dr*CD40, anti-*Dr*IgM, anti-*Dr*MHC-II, and anti-*Dr*IgZ used in this study, were prepared in our laboratory (21–24).

### Magnetic Sorting (MACS)

For the MACS of γδ T cells, blood, spleen, and head kidney tissues were collected in ice-cold Ca^2+^/Mg^2+^-free HBSS with heparin (10 U/mL). Single-cell suspensions of the spleen and head kidney were prepared by gently teasing the tissues through an 80-μm nylon mesh filter. Leukocytes were enriched from the cell suspensions through Ficoll-Hypaque (1.080 *g*/mL) centrifugation at 2,500 rpm for 25 min at room temperature and washed with ice-cold Ca^2+^/Mg^2+^-free HBSS. The γδ T cells were subjected to MACS, as described in a previous study (21). In brief, the cell suspension was blocked with 5% normal goat serum for 15 min at 10°C, incubated with anti-γ or anti-δ for 15 min at 10°C, washed with MACS buffer (PBS containing 2 mM EDTA and 0.5% BSA), and incubated for 15 min at 10°C with anti-IgG magnetic beads (Miltenyi Biotec). The cell suspension was applied to an LS separation column in accordance with the manufacturer’s instructions. Positive cells were cultured in L-15 medium (Life Technologies) supplemented with 10% FBS (Life Technologies), 100 U/mL penicillin, and 100 µg/mL streptomycin at 28°C overnight to detach the magnetic beads. The purity of the sorted γδ T cells was detected through RT-PCR and flow cytometry (FCM). In addition, αβ T cells for control purpose were sorted by similar protocol with the exception of incubating cells with anti-α or anti-β Abs instead of anti-γ or anti-δ Abs.

### FCM Analysis

The cells for FCM analysis were blocked with 5% normal goat serum for 1 h at 4°C and then incubated with the corresponding primary Abs, and non-specific mouse or rabbit IgG served as an isotype control. Afterward, the cells were washed and further incubated with secondary Abs, namely, PE-conjugated goat anti-mouse IgG and FITC-conjugated goat anti-rabbit IgG, for 1 h at 4°C. Fluorescence signals were determined by using a FACScan flow cytometer (BD Bio-sciences) at 488 nm. FCM analysis was based on forward/side scatter (FSC/SSC) characteristics and PE/FITC-conjugated fluorescence with CellQuest program as previously described (11, 25). At least 10,000 events were collected from the lymphocyte gate.

### Immunofluorescence Staining

For sorted cell staining, the cells were fixed with 2% paraformaldehyde for 10 min at room temperature, blocked with 5% normal goat serum and incubated with primary Abs (rabbit anti-γ, anti-β, anti-*Dr*CD4, anti-*Dr*CD8; mouse anti-δ, anti-α) at 4°C for 1 h. In parallel, the non-related Abs (rabbit IgG and mouse IgG) were used as negative controls. For intestine staining, cryosections were prepared and fixed for 5 min in 95% ethyl alcohol before incubating with primary Abs. Background autofluorescence was eliminated by treatment of cryosections for 10 min with 0.1 M glycine (pH 2.3). After washing, cells or intestine tissues were incubated with secondary Abs (PE-conjugated goat anti-mouse IgG and FITC-conjugated goat anti-rabbit IgG) at 4°C for 1 h. Additional staining with DAPI was performed before photomicrography. Samples were photographed under a two-photon laser-scanning microscope (Zeiss LSM710 NLO; Carl Zeiss, Oberkochen, Germany) at ×630 magnification.

### Electron Microscopy

The sorted γδ T cells were collected and fixed in 2.5% glutaraldehyde overnight, washed with 0.1 M PBS (pH 7.4) thrice, and post-fixed with 1% osmium tetroxide for 1 h. For scanning electron microscopy (SEM), the cells were washed with PBS and dehydrated in graded acetone. The isoamyl acetate was used to replace the acetone before drying the sample. Then, samples were coated with a layer of gold before observation under SEM (HITACHI S-3000N) (26). For transmission electron microscopy (TEM), cells were embedded in graded Epon 812 after being washed with PBS and dehydrated in graded acetone and kept at 60°C for 2 days. Thin (50–70 nm) sections were prepared and stained with uranyl acetate (2%) and lead citrate before observation under TEM (PHILIPSTECNAI10) (27).

### Western Blot Analysis

Freshly dissected zebrafish spleen and head kidney tissues and sorted γ^+^ or δ^+^ cells (4 × 10^4^) were added to cold RIPA buffer. Samples were homogenized and incubated for 30 min on ice. Cell lysates were centrifuged at 10,000 *g* for 10 min at 4°C. The protein content was quantified using the Bradford assay. Whole protein extracts or recombinant proteins were added to Laemmli loading buffer and incubated at 99°C for 10 min. Samples were separated by 12% SDS-PAGE under reducing conditions. After blotting onto PVDF membranes, 5% skimmed milk was used for blocking, and the membranes were incubated with primary Abs (anti-γ, anti-δ, anti-α, anti-β, anti-CD8α), followed by a secondary HRP-conjugated anti-rabbit/mouse IgG Ab (1:8,000). Immunoreactive proteins were visualized using a chemiluminescent immunodetection system (Tanon 4500).

### Tissue Distribution Analysis

Tissue distribution of γδ T cells in zebrafish was analyzed by the expression of *Dr*TRGC and *Dr*TRDC genes and the proportion of γ^+^/δ^+^ cells in tissues by real-time RT-PCR and FCM. For real-time RT-PCR, total RNAs were isolated from various tissues, including the head kidney, gill, spleen, liver, intestine, peripheral blood leukocyte (PBL), skin, heart, muscle, and brain. All PCR reactions were performed in a total volume of 10 µL by using a SYBR Premix Ex Taq kit (Takara Bio). The relative expression levels were calculated using the 2^−^ΔΔ^CT^ method with β-actin for normalization. Each PCR trial was performed in triplicate and repeated independently at least thrice. For FCM, leukocytes were isolated from the representative immune-relevant tissues, including the spleen, head kidney, skin, gill, intestine, and peripheral blood. The percentages of γ^+^/δ^+^ cells in these tissues were detected by FCM as described above.

### Phagocytosis Assay

Freshly isolated γδ T cells (4 × 10^4^) were incubated at 28°C with FITC-conjugated keyhole limpet hemocyanin (FITC-KLH; Sigma-Aldrich), red fluorescent latex beads (1 µm; Sigma-Aldrich, L-2778) or FITC-labeled *Aeromonas hydrophila* (A.h) at a cell/bead ratio of 1:10. Cells in the control group for active phagocytosis were incubated on ice. After 4 h, trypan blue (200 µg/mL) was added to quench the fluorescence of KLH/beads/A.h that had not been internalized for 5 min at 4°C. In parallel, γδ T cells incubated with FITC-KLH, red fluorescent beads, and FITC-A.h (28°C for 4 h) in the presence of cytochalasin B (80 µg/mL; Sigma-Aldrich) were set as controls. Then, cells were washed thrice with PBS before FCM analysis.

### Function of γδ T Cells in CD4^+^ T Cell Activation *In Vitro*

At 5 days before sacrifice, the fish (3–12 month) were immunized by i.p. injection with 10 µg KLH in combination with 10 ng LPS or A.h (2 × 10^7^ CFU/fish). Subsequently, Ag-stimulated CD4^+^ T cells were magnetically sorted from the blood, spleen, and head kidney, then stained with 5 µM CFSE (Beyotime) for 8 min at room temperature. The reaction was terminated by supplementing with 10% FBS (in l-15 medium), and cells were washed thrice with the medium. The primary γδ T cells were isolated from untreated fish and fish stimulated with 10 µg KLH (plus 100 ng LPS or 2 × 10^7^ CFU A.h) for 8 h and washed thrice to eliminate non-ingested Ags. In the Ag-presentation inhibition control group, γδ T cells were pretreated with chloroquine (80 µM, Sigma-Aldrich) for 1 h and then incubated with Ags as described above. In the cross-stimulation control group, the KLH-pulsed γδ T cells were co-cultured with CFSE-labeled CD4^+^ T_A.h_ cells. The T cell inhibitor cyclosporine A (CsA, 0.1 µg/mL; Sigma-Aldrich) was used as a control. After 3 days of co-culture, the cells were labeled with primary mouse anti-CD4 Ab and secondary PE-conjugated anti-mouse IgG Ab. The proliferation of CD4^+^ T cells was examined through FCM based on the division of CFSE-labeled cells in PE-CD4^+^ cell gate and analyzed by ModFit LT. The sample number for each group of fish exceeded 30, and all the experiments were conducted independently at least three times. The activation of CD4^+^ T cells was determined on the basis of the upregulation of Lck and CD154 through RT-PCR (21).

### Function of γδ T Cells in CD4^+^ T Cell Activation *In Vivo*

The fish were injected i.p. thrice with rabbit anti-γ/anti-δ or non-specific rabbit IgG (as control) at a dose of 10 µg/fish, followed by administration of Ag (10 µg KLH plus 10 ng LPS). At 3 days after the last Ag stimulation and Ab administration, the deletion level of γδ T cells was examined through FCM. The activation of CD4^+^ T cells was evaluated by the percentage of CD4^+^CD154^+^ T cells in leukocytes from the peripheral blood, spleen, and head kidney, as determined using mouse anti-*Dr*CD4 and rabbit anti-*Dr*CD154 primary Abs followed by FITC- and PE-conjugated anti-rabbit IgG and anti-mouse IgG secondary Abs through FCM. As a downstream event of CD4^+^ T cell activation, the B cell activation was further examined on the basis of the percentage of mIgM^+^CD40^+^ B cells in leukocytes from the peripheral blood, spleen, and head kidney, as determined using mouse anti-*Dr*IgM and rabbit anti-*Dr*CD40 primary Abs followed by FITC- and PE-conjugated anti-rabbit IgG and anti-mouse IgG secondary Abs. The expression of Lck, CD154, mIgM, and CD40 was determined to evaluate the activation of CD4^+^ T cells and B cells through real-time PCR (11).

### Function of γδ T Cells in Ab Production

Before the i.p. immunization with KLH (10 µg/fish) together with CFA, zebrafish were administered with anti-γ/δ, or anti-α/β and IgG (10 µg/fish) or CsA (5 µg/fish) thrice at a 12 h time interval. The control groups received CFA in combination with mock PBS. The second immunization was given at 14 days. After 28 days of immunization, the serum and intestinal mucus were collected, the latter of which was dissolved in PBS (pH 7.2) containing protease inhibitors [1 × protease inhibitor cocktail (Roche), 1 mM phenylmethylsulfonyl fluoride (PMSF, Sigma), 0.1 mg/mL soybean trypsin inhibitor (Sigma), and 0.5% BSA (Sigma)] and centrifuged at 400 *g* for 10 min to remove cell debris as previously described (28). The IgM and IgZ Abs against KLH were measured by ELISA (22). Ab titer is defined as the highest serum or mucus dilution at which the A_450_ ratio (A_450_ of postimmunization sera/A_450_ of preimmunization sera) is greater than 2.1.

### Adoptive Transfer Assays

Three days before each immunization at days 1 and 14, the recipient fish were continuously injected with rabbit anti-*Dr*MHC-II Ab thrice with a 12 h interval to eliminate APCs. In the same way, αβ T cells of some recipient fish were eliminated by administering mouse anti-α and rabbit anti-β thrice simultaneously. γδ T cells were then magnetically sorted from the untreated fish and exposed to KLH (10 µg plus 100 ng LPS) for 8 h. The cells in the control group were treated with mock PBS. The γδ T cells were washed thrice with L-15 medium to remove the free Ag and adjusted to 1 × 10^6^ cell/mL, 1 × 10^7^ cell/mL, and 1 × 10^8^ cell/mL. Afterward, 10 µL of cell suspensions was i.p. injected into the recipient fish in different treatments along with the immunization with KLH.

### Statistical Analysis

All data are presented as the mean ± SD of each group. Statistical evaluation of differences between means of experimental groups was done by Student’s *t* tests. Statistical significance was considered at *P* < 0.05 or *P* < 0.01. The sample number for each group of fish exceeded 10. All experiments were replicated at least three times.

## Results

### Characterization of the Constant Regions of Zebrafish TCR-γ and TCR-δ

Because the TCR-γ/TCR-δ complex is a hallmark of γδ T subset, we attempted to identify γδ T cells in zebrafish using anti-zebrafish TCR-γ and TCR-δ Abs. To produce these Abs, the cDNAs encoding the constant regions of zebrafish TCR-γ (*Dr*TRGC) and TCR-δ (*Dr*TRDC) were identified for the design of antigen epitope peptides. The *Dr*TRGC and *Dr*TRDC cDNAs were cloned from spleen and intestinal tissues by using primers designed on the basis of the predicted sequences in NCBI (AY973884.1 and CAK05280.1). The cDNA of *Dr*TRGC is 540 bp in length, with a 537 bp ORF that encodes 179 amino acids (GenBank accession number KX009744). The cDNA of *Dr*TRDC is 471 bp in length, with a 468 bp ORF that encodes 156 amino acids (GenBank accession number KX009743; Figure S1 in Supplementary Material). The organizations of *Dr*TRGC and *Dr*TRDC genes were clarified by comparing the cDNAs of *Dr*TRGC and *Dr*TRDC with the corresponding genomic sequences. *Dr*TRGC and *Dr*TRDC genes contain three exons and two introns located within 3.09 and 1.05 kb genomic fragments on chromosome 2, respectively (Figure S2A in Supplementary Material). The genes adjacent to *Dr*TRGC and *Dr*TRDC clusters were retrieved by using Genscan and BLAST programs. According to the genes around the TRGC cluster on human chromosome 7 or mouse chromosome 13, *epdr1, stard3nl, amph*, and *hecw1* genes were clustered on zebrafish chromosome 2, and they shared a highly conserved chromosome synteny with their human counterparts; however, the synteny of these genes was in converse orders between mice and humans (Figure S2B in Supplementary Material). Similarly, *hnrnpc, chd8, tox4, sall2*, and *TRA* around the TRDC clusters on human and mouse chromosomes 14 were also clustered around the *Dr*TRDC loci on chromosome 2, although the synteny of these genes was in converse orders between zebrafish and humans or mouse (Figure S2B in Supplementary Material).

*Dr*TRGC and *Dr*TRDC proteins were predicted as membrane molecules with molecular weights of ~20 and ~17 kDa, respectively. Both proteins exhibit hallmark TCR features: an immunoglobulin constant (Ig-C) domain (107 aa for *Dr*TRGC and 103 aa for *Dr*TRDC), a connecting peptide of varying lengths (38 aa for *Dr*TRGC and 23 aa for *Dr*TRDC), a transmembrane region (22 aa for *Dr*TRGC and 20 aa for *Dr*TRDC), and a cytoplasmic domain (11 aa for *Dr*TRGC and 10 aa for *Dr*TRDC). Multiple alignments show that the key functional amino acid residues in *Dr*TRGC and *Dr*TRDC proteins share a higher degree of homology with that of other species, which including C^33^ (first-Cys), P^40^, W^47^, C^93^ (second-Cys) in the Ig-C domain of TRGC; Y^172^, K^178^ in the transmembrane region of TRGC; P^9^, C^33^ (first-Cys), F^38^, Y^73^, C^92^ (second-Cys) in the Ig-C domain of TRDC, and R^156^, K^161^ in the transmembrane region of TRDC (Figure S3 in Supplementary Material). C^33^ and C^93^ in *Dr*TRGC and C^33^ and C^92^ in *Dr*TRDC of the Ig-C domains, which are involved in an intra-chain disulfide bond, are highly conserved from fish to mammals. Another conserved cysteine is present in the connecting peptides of *Dr*TRGC (C^129^) and *Dr*TRDC (C^128^). This cysteine likely forms an inter-chain disulfide bridge between the connecting peptides of TRGC and TRDC in mouse models. Moreover, a characteristic CX6PX6WX45C motif is found in the Ig-C domain of TRGC in all species. *Dr*TRGC and *Dr*TRDC, respectively, share 34–61% and 34–39% amino acid similarities with those of their mammalian counterparts. Phylogenetic analysis revealed that *Dr*TRGC and *Dr*TRDC were clustered independently from those of other fish and then merged with mammalian counterparts to form a solitary branch (Figure S4 in Supplementary Material). These findings demonstrated the molecular and functional conservations of TRGC and TRDC homologs from fish to mammals during vertebrate evolution.

### Identification of γδ T Cells from Zebrafish

Based on the above predicted structural information, the potential immunogenic epitope sequences of *Dr*TRGC (a 17-aa peptide, S^123^–Q^139^) and *Dr*TRDC (a 14-aa peptide, S^12^–C^25^) chosen for immunization were located on the surface of a more exposed β-barrel structure in the extracellular Ig-C domains of the two molecules (Table S2 in Supplementary Material). Abs against *Dr*TRGC and *Dr*TRDC (namely anti-γ and anti-δ) were produced based on these epitope peptides from rabbit or mouse, respectively. ELISA demonstrated that the prepared Abs yielded an average titer of more than 1:10,000. Western blot revealed that the affinity-purified Abs have high specificities to the antigen proteins, as determined by their specific binding to the recombinant *Dr*TRGC and *Dr*TRDC proteins prepared from *E. coli* and to the corresponding endogenous proteins from spleen and head kidney tissues. To further demonstrate specificity, blocking peptides were also included in the WB analysis. Results showed the band signals weakened or disappeared when incubating with blocking peptides together with their corresponding Abs (Figures S5A–C in Supplementary Material). Minimal cross-reactions were detected between other TCRs (Figure S5A in Supplementary Material). In addition, three other Abs against zebrafish TCR-α, TCR-β, and CD8α (namely anti-α, anti-β, and anti-CD8) required for experiment were also produced based on their epitope sequences in the extracellular domains of *Dr*TRAC, *Dr*TRBC, and CD8α, respectively (Table S2 in Supplementary Material).

**Figure 1:**
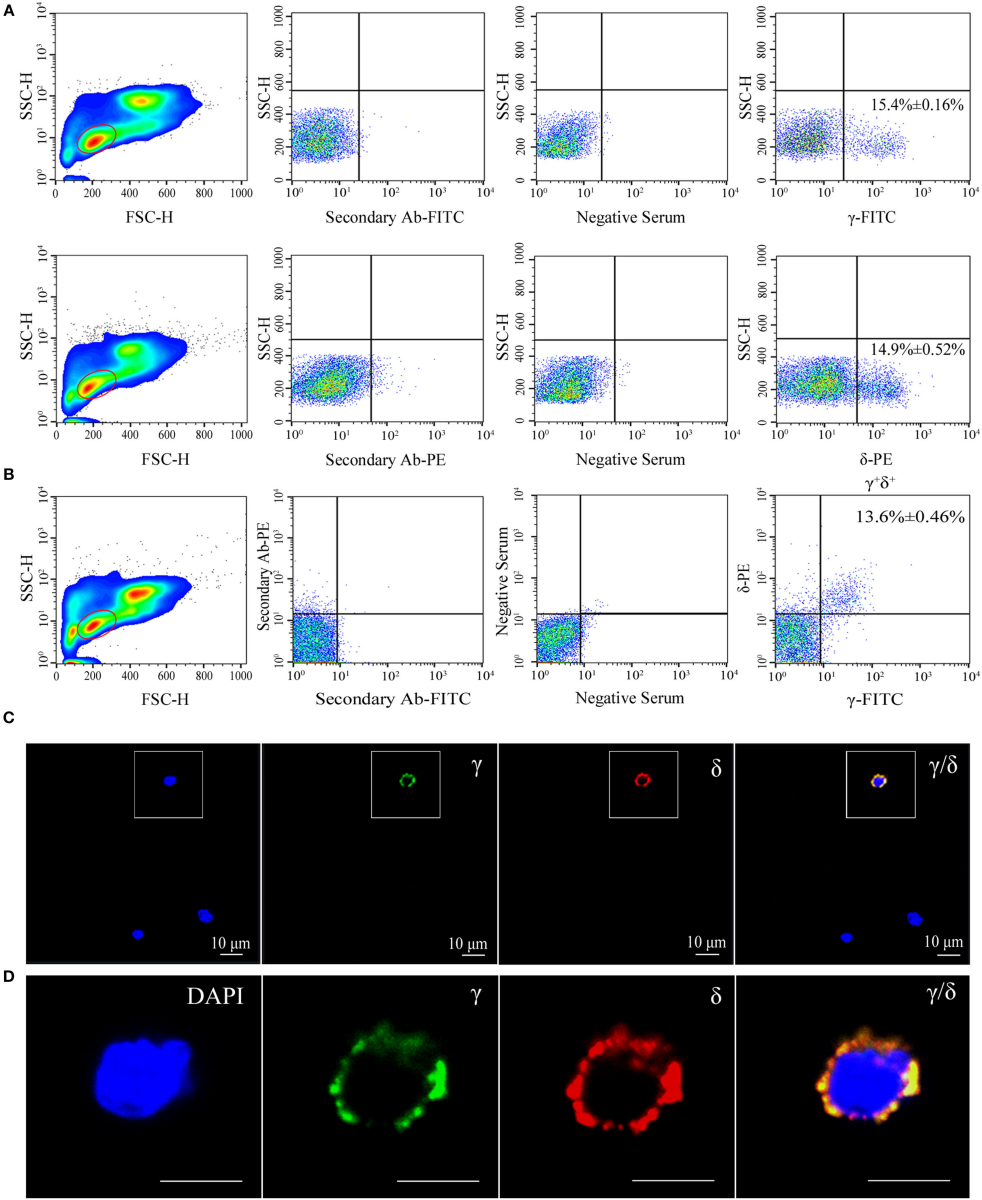
**Flow cytometry (FCM) and immunofluorescence analyses of γ^+^δ^+^ cells in zebrafish**. **(A)** FCM detection of γ and δ single-positive (γ^+^ and δ^+^) cells in leukocytes from zebrafish peripheral blood, head kidney, and spleen tissues, respectively. The left panels show FSC/SSC analyses and the red-outlined gates represent lymphoid cells. Middle panels represent the cells treated only with the secondary Ab (FITC-conjugated goat anti-rabbit IgG or PE-conjugated goat anti-mouse IgG) and with the preimmunization serum. The right panels represent staining of cells with rabbit anti-γ or mouse anti-δ Abs. **(B)** FCM detection of γδ-double positive (γ^+^δ^+^) cells in leukocytes from zebrafish peripheral blood, head kidney, and spleen tissues. FSC/SSC analyses are shown in the left panel and red-outlined gate represents lymphoid cells. The middle panel shows the analysis of cells incubated only with secondary Abs and with the preimmunization serum. The right panel represents double staining of cells with rabbit anti-γ and mouse anti-δ Abs. **(C)** Confocal microscopy image of γ^+^δ^+^ cells stained with rabbit anti-γ and mouse anti-δ Abs. Non-related Abs, including mouse IgG and rabbit IgG, were used as negative controls (data not shown). DAPI stain showed the locations of the nuclei. A laser-scanning confocal microscopy (Zeiss LSM-710) was used in the analyses (original magnification ×630, scale bar, 10 µm). **(D)** Enlarged images of γ^+^δ^+^ cells in the white-outlined square of E. Scale bar, 5 µm.

**Figure 2:**
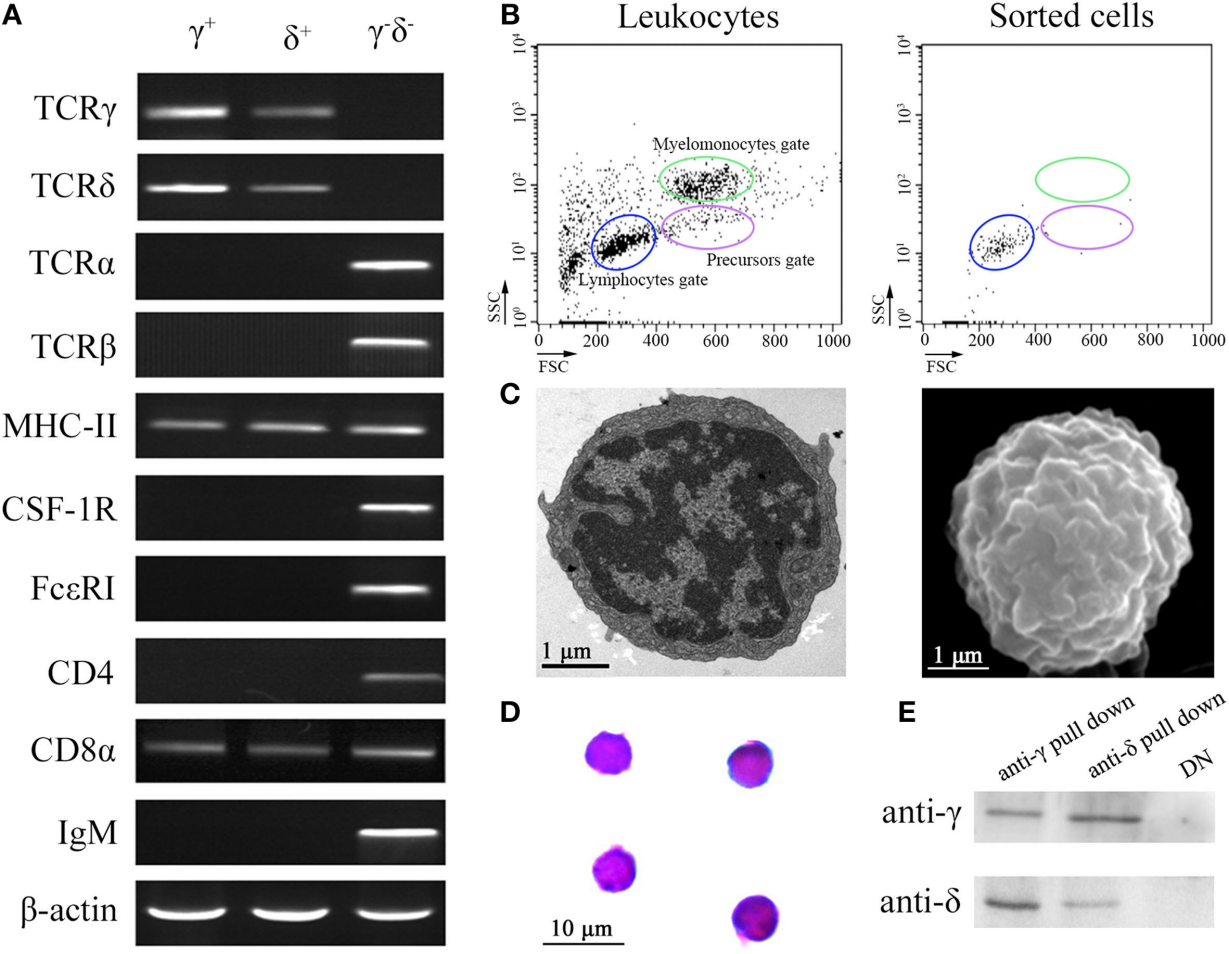
**Cellular and molecular identification of zebrafish γδ T cells**. **(A)** RT-PCR assay analyzed the expression of T cell markers (γ, δ, α, β, CD4, and CD8), antigen-presenting cell marker (MHC-II), B cell marker (mIgM), and myeloid cell (monocyte/macrophage and dendritic cell) markers (CSF-1R and FcεRI). The expressions of these markers from negative selection cells were used as control. **(B)** FCM analyzed the FSC/SSC profiles of leukocytes and the sorted cells. **(C)** Transmission electron microscopy and scanning electron microscopy show the detailed morphologies of sorted γδ T cells. Scale bar 1 µm (bottom left). **(D)** Wright-Giemsa staining indicates the morphologies of the sorted γδ T cells. Scale bar 10 µm (bottom left). Original magnification ×1,000. **(E)** Detect the expressions of γ and δ in the anti-γ and anti-δ pull down cells, respectively, by Western blot.

**Figure 3:**
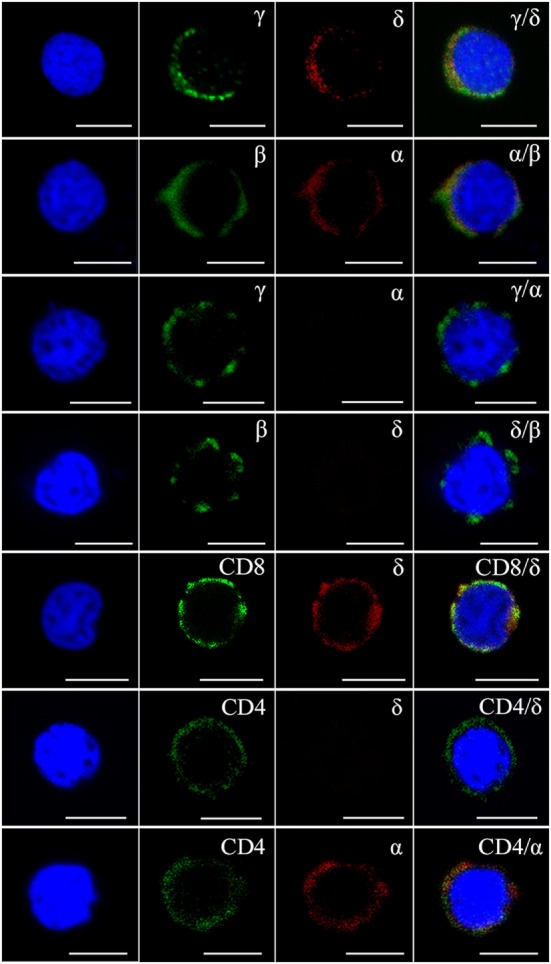
**Immunophenotype analysis of zebrafish γδ T cells by Abs against various surface molecules**. The magnetic sorted cells were double stained with anti-γ, anti-δ, anti-α, anti-β, anti-CD8, and anti-CD4 Abs in different combinations (rabbit anti-γ/mouse anti-δ, rabbit anti-β/mouse anti-α, rabbit anti-γ/mouse anti-α, rabbit anti-β/mouse anti-δ, rabbit anti-CD8/mouse anti-δ, rabbit anti-CD4/mouse anti-δ, and rabbit anti-CD4/mouse anti-α, respectively). Positive control cells were stained with rabbit anti-β and mouse anti-α Abs. Non-related Abs, including mouse IgG and rabbit IgG, were used as negative controls (data not shown). DAPI stain showed the locations of the nuclei. A laser-scanning confocal microscopy (Zeiss LSM-710) was used in the analyses (original magnification × 630, scale bar, 5 µm).

Flow cytometry analysis of leukocytes from peripheral blood, head kidney, and spleen tissues using rabbit anti-γ or mouse anti-δ Ab alone showed a distinct γ or δ single-positive cell population (γ^+^ or δ^+^) with a similar proportion of the total lymphoid cells (15.4 or 14.9%), respectively (Figure 1A); and it also showed a distinct γ and δ double-positive subset (γ^+^δ^+^) with a similar proportion (13.6%) seen in γ or δ single-positive population by co-staining the leukocytes with rabbit anti-γ and mouse anti-δ Abs (Figure 1B). These γ^+^δ^+^ cells were also clearly detected in mixed leukocytes by double-immunofluorescence staining under a laser-scanning microscopy (Figures 1C,D). These γ^+^δ^+^ cells were further sorted from the mixed leukocytes by anti-γ or anti-δ Ab-coated magnetic beads and subjected to cellular and molecular identification. Beforehand, the forward/side scatter (FSC/SSC) characteristics of the mixed leukocytes were examined through FCM, and result showed that three distinct scatter populations can be resolved by FSC/SSC analysis, whose profile was closely matched with those obtained from whole kidney marrow, in which major blood cell linages of lymphocytes (FSC^int^SSC^lo^), precursors (FSC^hi^SSC^int^), and myelomonocytes (FSC^hi^SSC^hi^) were included (25). Then, the sorted γ^+^δ^+^ cells were subjected back to FSC/SSC analysis. Result showed that nearly all cells were enriched in the lymphocyte scatter population (FSC^int^SSC^lo^ gate), and they showed typical lymphocyte morphology by Wright-Giemsa staining (Figures 2B,D). Next, gene expression analysis was performed to show the independence of the sorted γ^+^δ^+^ subset from other cells by using a series of cellular hallmarks, including APC marker (MHC-II), T cell markers (γ, δ, α, β, CD4, and CD8), B cell marker (mIgM), and myeloid cell (monocyte/macrophage) markers (CSF-1R and FcεRI), through RT-PCR. Result showed that either γ^+^ or δ^+^ cells can express both δ and γ transcripts; however, neither γ^+^ nor δ^+^ cells express α, β, mIgM, CSF-1R, and FcεRI transcripts. This finding excludes α^+^β^+^ T cells, mIgM^+^ B cells, and monocytes/macrophages from the sorted γ^+^δ^+^ cells (Figure 2A). Moreover, immunoprecipitation and Western blot analyses further confirmed the expression of δ and γ at protein levels in the sorted γ^+^ or δ^+^ cells (Figure 2E), which also providing observation that γ and δ proteins co-localized on γ^+^δ^+^ cells by the cross reactivity between the two proteins. In addition, cross double-immunofluorescence staining in γα and δβ combinations showed few γα and δβ double-positive (γ^+^α^+^ and δ^+^β^+^) cells could be detected in the sorted γ^+^δ^+^ cells, which indicated that zebrafish γδ T cell is a distinct subset independent of αβ T cells. Double-immunofluorescence staining by δCD8 and δCD4 showed considerable γ- and δ-positive cells exhibit CD8-positive (δ^+^CD8^+^) and few δCD4 double positive (δ^+^CD4^+^) existed (Figure 3). Detailed morphology of the γ^+^δ^+^ cells were detected at approximately 5 µm across, with round dense nuclei surrounded by a thin round ring of cytoplasm and numerous surface frills, as determined by TEM and SEM (Figure 2C). Finally, we analyzed the expression levels of *Dr*TRGC and *Dr*TRDC in different tissues and found that *Dr*TRGC and *Dr*TRDC were widely distributed in various lymphoid-associated tissues, such as spleen, head kidney, skin, gill, intestine, and PBLs, which was consistent with the performance of the lymphoid cells (Figure S6 in Supplementary Material). FCM results showed the γ^+^δ^+^ T cell population consisted of 7.7, 9.3, 16.9, 18.3, and 20.5% of the total lymphocytes in the spleen, head kidney, skin, gills, and intestinal tissues, respectively (Figure S7 in Supplementary Material). These results strongly indicated the existence of γδ T cell lineage in zebrafish, which exhibits phenotypic and morphological similarities to mammals.

### Phagocytic Capacity of Zebrafish γδ T Cells

**Figure 4:**
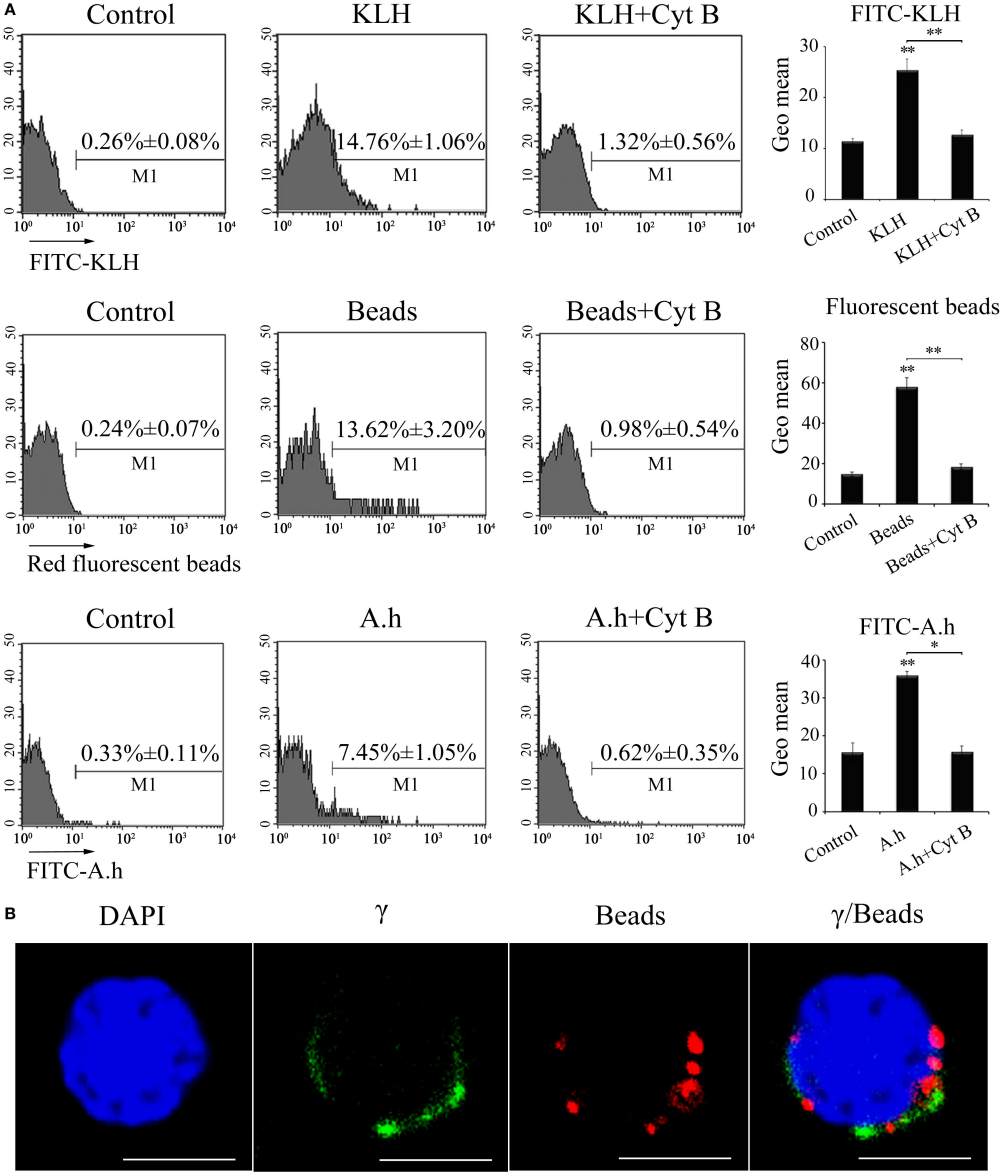
**Phagocytic ability of zebrafish γδ T cells**. **(A)** FCM detected the phagocytic ability. γδ T cells were magnetically sorted and incubated with FITC-KLH, 1 µm red fluorescent latex beads or FITC-A.h at 28°C for 4 h. Cells in control group for active phagocytosis were incubated in ice. In parallel, γδ T cells incubated with FITC-KLH, red fluorescent beads, and FITC-A.h (28°C for 4 h) in the presence of cytochalasin B were set as controls. The numbers above the marker bars in each panel indicate the percentages of phagocytic γδ T cells. The geometric means of the fluorescence intensities computed from the outlined region represent the phagocytic ability of γδ T cells in each treatment group. Means ± SD of three independent experiments are shown. *P < 0.05, **P < 0.01. **(B)** Confocal microscopy of 1 µm red fluorescent latex beads by γδ T cells. DAPI stain showed the location of the nuclei. Original magnification ×630. Scale bar, 5 µm.

The phagocytic capacity of γδ T cells was initially examined through FCM to evaluate the potential ability of zebrafish γδ T cells acting as a kind of APCs in priming adaptive immunity. The γδ T cells were magnetically sorted from the selected PBLs, spleens, and head kidney tissues and then examined to share a high degree of purity (>95%) through FCM. The cells were incubated with FITC-KLH, 1 µm red fluorescent latex beads (Sigma L2778-1ML), or FITC-A.h at 28°C. The percentages of the phagocytic γδ T cells reached 14.76 ± 1.06% (for FITC-KLH), 13.62 ± 3.20% (for latex beads), and 7.45 ± 1.05% (for FITC-A.h), which were significantly higher than the 0.26 ± 0.08%, 0.24 ± 0.07%, and 0.33 ± 0.11% for the corresponding control groups at 4°C. Cytochalasin B treatment greatly inhibited FITC-KLH, latex beads, and FITC-A.h uptake by the γδ T cells. This finding indicated the γδ T cells could phagocytose substances in an actin polymerization-dependent manner. The enhanced amounts of Ag phagocytosis by γδ T cells were also determined on the basis of the mean fluorescence intensity of FITC and red fluorescence (Figure 4A). Immunofluorescence confirmed that the red beads were indeed inside the cells (Figure 4B). Thus, zebrafish γδ T cells exhibit potent non-specific phagocytic abilities for both soluble and particulate antigens.

### Function of Zebrafish γδ T Cells in CD4^+^ T Cell Activation *In Vitro*

**Figure 5:**
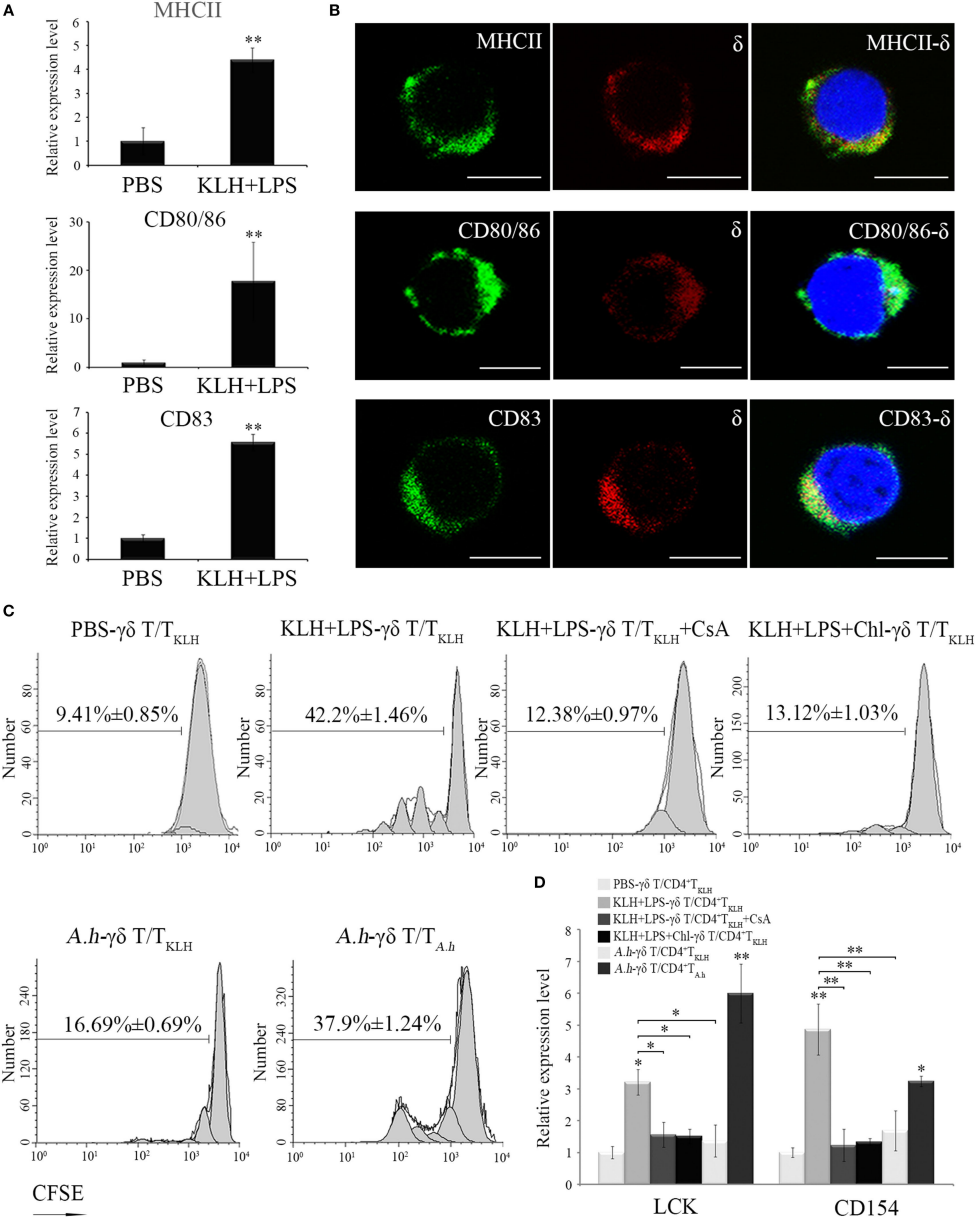
***In vitro* evaluation of Ag-presenting capacity of zebrafish γδ T cells**. **(A)** Real-time PCR analyses of the expression levels of MHC-II, CD80/86, and CD83 in PBS- and KLH, plus LPS-loaded γδ T cells *in vitro*. **(B)** Immunofluorescence staining of δ^+^MHC^+^, δ^+^CD80/86^+^, and δ^+^CD83^+^ cells. Leukocytes were stained with mouse anti-δ and rabbit anti-MHCII, CD83, and CD80/86, respectively. Non-related Abs, including mouse IgG and rabbit IgG, were used as negative controls (data not shown). DAPI stain showed the locations of the nuclei. Original magnification ×630. Scale bar, 5 µm. **(C)** The proliferation and activation of CD4^+^ T_KLH_ cells primed by Ag-loaded γδ T cells were determined after CFSE dilution and measured *via* FCM. The proliferation of CD4^+^ T_KLH_ cells primed by mock PBS-treated γδ T cells served as a negative control. Cyclosporine A was used for the T cell inhibitor control. For the Ag-presentation inhibition control, γδ T cells was pretreated with Chloroquine for 1 h and then incubated with Ags. In cross-stimulation control group, the KLH-pulsed γδ T cells were co-cultured with CFSE-labeled CD4^+^ T_A.h_ cells. The numbers above the marker bars indicate the percentage of CFSE-diluted cells in each panel. Data represent three independent experiments. **(D)** The expression levels of Lck and CD154 were detected through real-time PCR. Means ± SD of three independent experiments are shown. *P < 0.05, **P < 0.01.

An *in vitro* antigen-specific CD4^+^ T cell activation assay was performed to investigate whether zebrafish γδ T cells can act as APCs to initiate adaptive immunity. The distribution of MHC-II, CD80/86, and CD83, three specific APC markers involved in Ag presenting and costimulatory signaling to CD4^+^ T cells, were determined in the activated γδ T cells magnetically sorted from the antigen (KLH)-stimulated fish through RT-PCR and double-immunofluorescence staining (Figures 5A,B). As expected, MHC-II, CD80/86, and CD83 were clearly expressed on the surfaces of the activated γδ T cells, and their expression was significantly upregulated in response to Ag stimulation (Figure 5A). This finding provides initial evidence that zebrafish γδ T cells exhibit the functional characteristics of APCs. For the T cell activation assay, the siblings generated from the same lineage after five generations of inbreeding were used to lessen the potential allogenic reactions as much as possible, because the pure inbred zebrafish lines are difficult to create due to inbreeding depression (21, 25, 29). The primary γδ T cells that were sorted from non-stimulated fish were boosted *in vitro* by KLH in combination with LPS, a pathogen-associated molecular pattern expected to provide a costimulatory signal, and then co-cultured with the KLH-stimulated CD4^+^ T cells (CD4^+^ T_KLH_) isolated from the fish *in vivo* administrated with KLH. Expectedly, KLH-loaded γδ T cells remarkably enhanced the cognate CD4^+^ T_KLH_ cell proliferation by approximately 4.5-fold compared with the mock PBS-loaded γδ T cell controls. Next, an Ag-presentation-inhibition assay and a cross-stimulation experiment were further performed to exclude the potential allogenic reactions. Results showed that CD4^+^ T_KLH_ cell response was remarkably decreased by incubating KLH-loaded γδ T cells with chloroquine, an inhibitor of endosomal and lysosomal acidifications and proteolyses required for MHC-II-mediated antigen presentation. *A.h*-pulsed γδ T cells elicited considerably less effects on the increase in CD4^+^ T_KLH_ proliferation than KLH-pulsed γδ T cells did. The CD4^+^ T_KLH_ cell proliferation was also significantly decreased by CsA, an inhibitor of thymus-dependent T cell activation (Figure 5C). These results suggested that the Ag-specific reaction largely contributes to the major proliferation of CD4^+^ T cells. Besides, the activation of CD4^+^ T_KLH_ cells stimulated by KLH-activated γδ T cells was also examined by the increased expression of Lck and CD154 (Figure 5D). Collectively, the above results demonstrated that zebrafish γδ T cells can act as powerful APCs.

### Function of Zebrafish γδ T Cells in CD4^+^ T Cell Activation *In Vivo*

**Figure 6:**
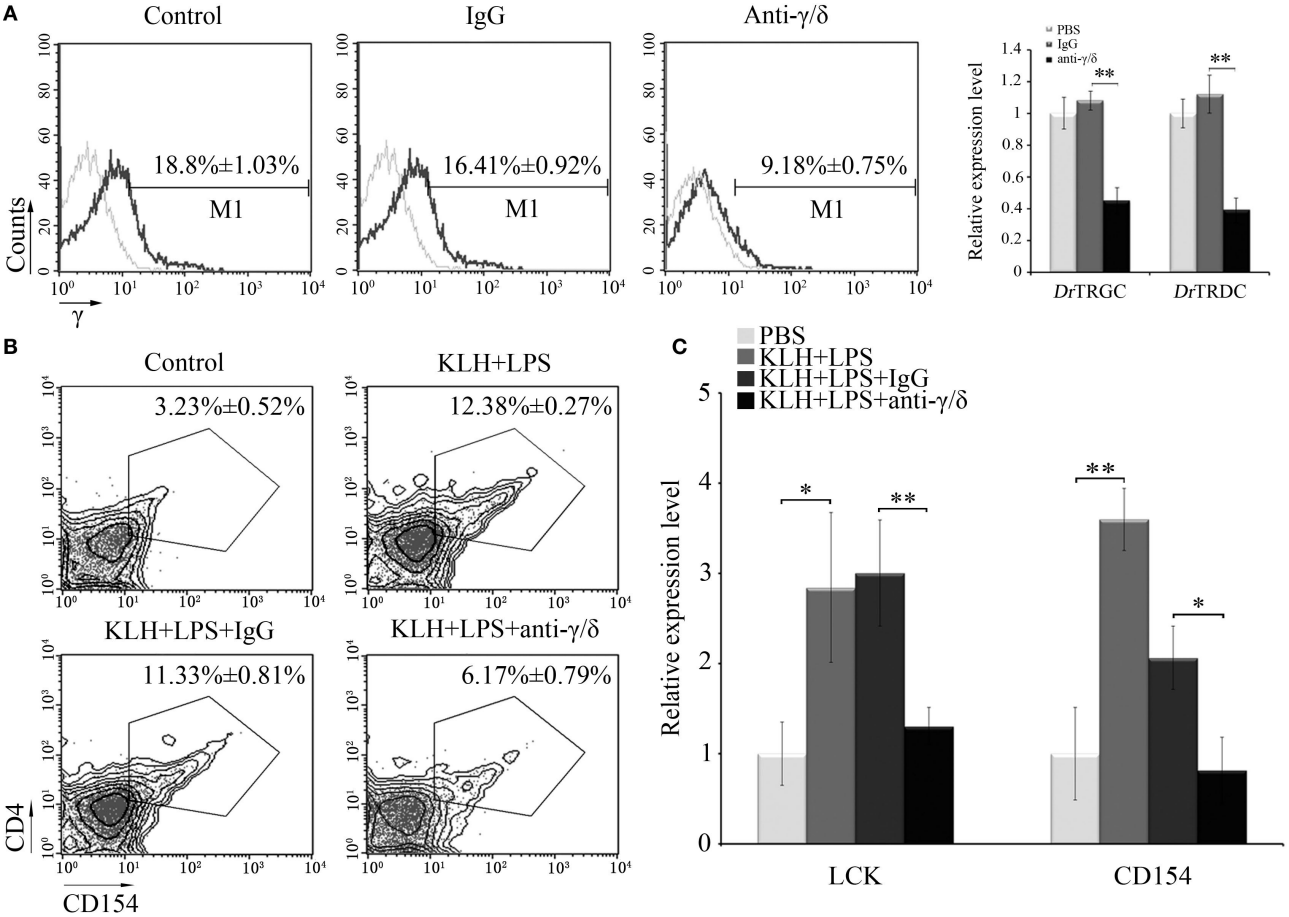
***In vivo* evaluation of Ag-presenting capacity of zebrafish γδ T cells**. **(A)** Effects of anti-γ and anti-δ Abs on *in vivo* depletion of γδ T cells were assessed by FCM and real-time PCR. Negative controls treated with PBS or normal rabbit IgG were created. Light gray graphs show the background staining with an irrelevant Ab on the target gated population. Numbers above the marker bars indicate the percentage of γδ T cells in each treatment group. Each result was obtained from 30 fishes. Means ± SD of three independent experiments are shown. *P < 0.05, **P < 0.01. **(B)** The degree of CD4^+^ T cell activation is represented by the percentage of CD4^+^CD154^+^ T cells in leukocyte population, determined by FCM. **(C)** The expression levels of Lck and CD154 were detected by real-time PCR. The numbers above the outlined areas in each block diagram indicate the average percentages of CD4^+^CD154^+^ T cells in each treatment group. In the real-time PCR assay, PCRs were run in combination with the endogenous β-actin control. Means ± SD of three independent experiments are shown. *P < 0.05, **P < 0.01.

An *in vivo* depletion assay was performed to obtain additional evidence supporting the involvement of zebrafish γδ T cells in priming Ag-specific CD4^+^ T cells. The fishes received anti-γ or anti-δ Abs, and the amounts of γδ T cells decreased by approximately 50% lower than those of the PBS- or non-related rabbit IgG-treated fishes (Figure 6A). Compared with that of the antigen (KLH)-immunized control groups, the proportion of the activated CD4^+^ T_KLH_ cells (CD4^+^CD154^+^ T_KLH_) decreased from 12.38 ± 0.27% to 6.17 ± 0.79% in the γδ T cell-depleted groups. Compared with those of the normal KLH-immunized groups, the percentages of CD4^+^CD154^+^T_KLH_ cells (11.33 ± 0.81%) did not significantly decrease in the non-related IgG-treated groups (Figure 6B). Similarly, the expression levels of Lck and CD154 upon KLH stimulation in PBLs and head kidney leukocytes (HKLs) were remarkably (*P* < 0.01) downregulated in the γδ T cell-depleted groups (Figure 6C). These results provided *in vivo* evidence that zebrafish γδ T cells play an essential role in Ag-specific T cell activation in the initiation of adaptive immunity.

### Function of Zebrafish γδ T Cells in B Cell Activation and IgM Production

**Figure 7:**
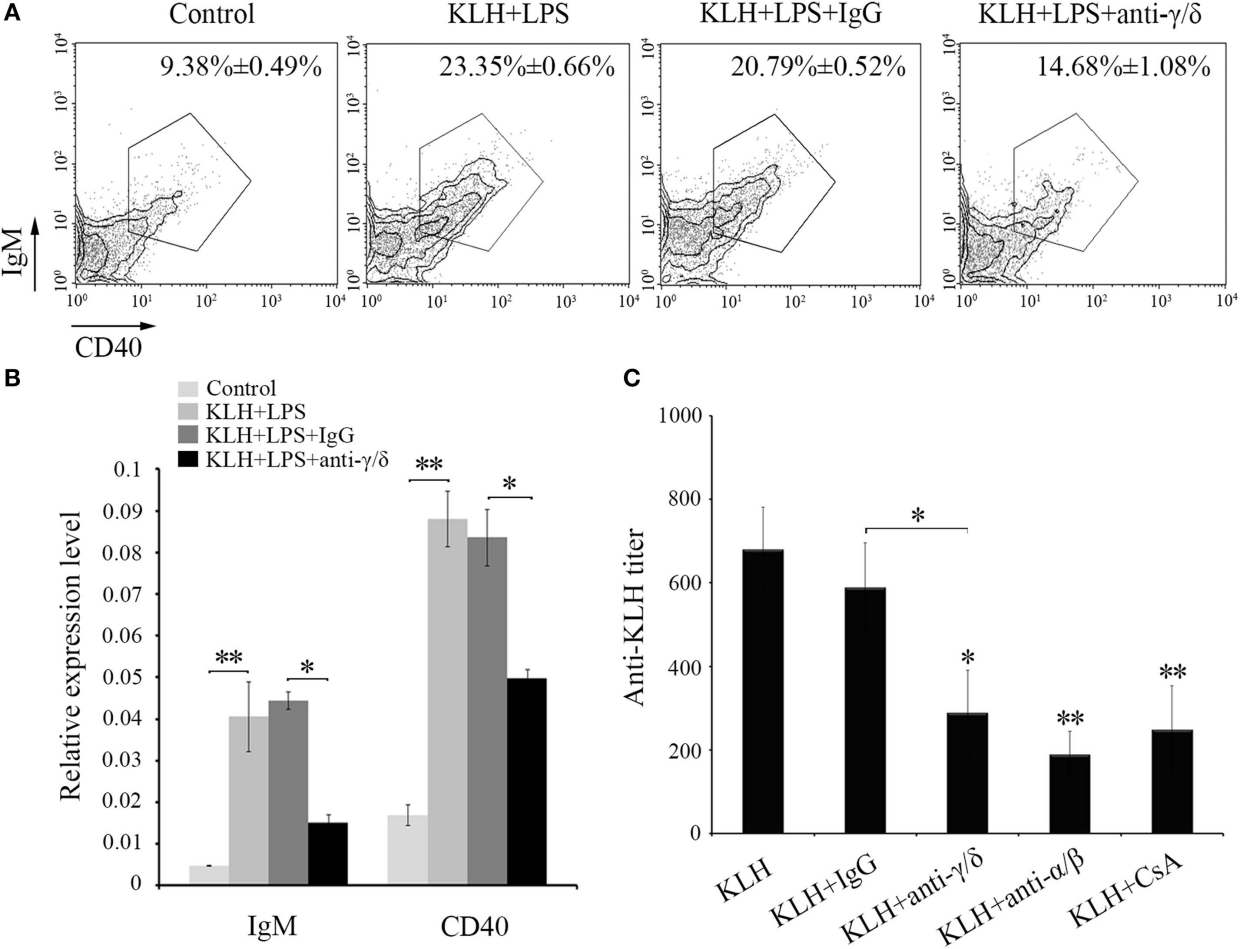
***In vivo* evaluation of zebrafish γδ T cells in B cell activation and IgM production**. The degree of B cells activation is represented by the percentage of mIgM^+^CD40^+^ B cells measured by FCM and by the expression levels of IgM and CD40 detected by real-time PCR **(A,B)**. In the flow cytometric analysis, different treatments are shown at the top of each block diagram. The data above the outlined area indicate the average percentages of mIgM^+^CD40^+^ B cells in each treatment group. In the real-time PCR assay, PCRs were run in combination with the endogenous β-actin control. Means ± SD of three independent experiments are shown. *P < 0.05, **P < 0.01. **(C)** The titers of IgM against KLH in each treatment group were examined by ELISA. The control groups immunized with KLH only. All of the groups were titered from 1/100 to 1/800, and the titer was ascertained based on the highest serum dilution at which the A450 ratio (A450 of postimmunization sera/A450 of preimmunization sera) is ≥ 2.1. The data of the background control group with PBS administration were subtracted from each experimental group.

**Figure 8:**
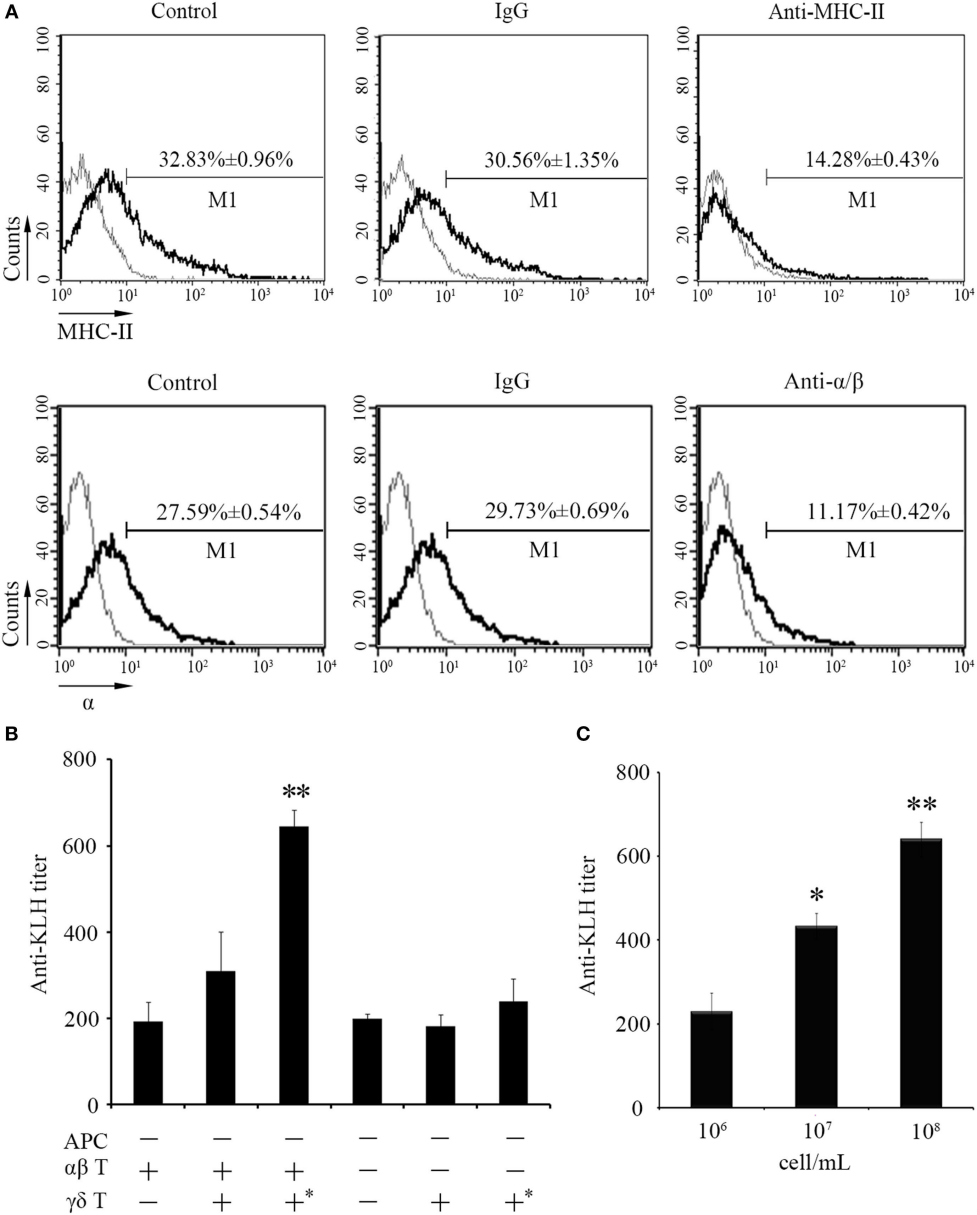
**An adoptive transfer assay of γδ T cells in the IgM production**. **(A)** The effect of anti-MHC-II Ab and anti-α/β Abs on *in vivo* depletion of antigen-presenting cells (APCs) and αβ T cells were assessed by FCM. Negative controls treated with PBS or normal rabbit IgG were created. Light gray graphs show the background staining with an irrelevant Ab on the target gated population. The numbers above the marker bars indicate the percentage of MHC-II^+^ APC and α^+^ T cells in each treatment group. Each result was obtained from 30 fishes. **(B)** ELISA for KLH-specific IgM Abs in the serum of fishes received adoptive transfer in different-treatment combinations. All the recipient fishes were administered with anti-MHC-II Ab to eliminate the basal MHC-II^+^ APCs. Double depletion of APCs and αβ T cells was performed by co-administration with anti-MHC-II Ab and anti-α/β Abs into fishes followed by the transfer of γδ T cells. The “APC−” group means recipient fishes with an efficient APC-depletion as shown in **(A)** (upper panel); the “αβ T−” group means recipient fishes with an efficient αβ T cell-depletion as shown in **(A)** (lower panel); the “αβ T+” group means recipient fishes with no depletion treatment; the “γδ T−” group means recipient fishes with no γδ T cell adoptive transfer; the “γδ T+” group means recipient fishes received adoptive transfer of non-activated γδ T cells; the “γδ T+*” group means recipient fishes received adoptive transfer of activated γδ T cells with antigen (KLH in combination of LPS) stimulation. These groups were assembled in different combinations as designated in the figure body for different experimental purpose. **(C)** ELISA for KLH-specific IgM Abs in the serum from the recipient fishes transferred with different dosages (10^6^, 10^7^, and 10^8^ cell/mL) of activated γδ T cells. All of the groups were titered from 1/100 to 1/800, and the titers were ascertained based on the highest serum dilution, at which the A450 ratio (A450 of postimmunization sera/A450 of preimmunization sera) is ≥ 2.1. The data of the background control group with PBS administration were subtracted from each experimental group.

Next, *in vivo* B cell activation and IgM production assays were performed for further determination of the functions of zebrafish γδ T cells in the initiation of adaptive immunity. Compared with those of the Ag-immunized normal group, the IgM^+^ CD40^+^ B cells in the γδ T cell-depleted group decreased from 23.35 ± 0.66% to 14.68 ± 1.08% when the cells were injected with KLH combined with LPS. By contrast, the percentage of IgM^+^CD40^+^ B cells did not significantly decrease in the non-related IgG-treated group compared with that in the normal immunized group (Figure 7A). The expression levels of IgM and CD40 in PBLs and HKLs were remarkably downregulated in the γδ T cell-depleted group (Figure 7B). The production of the systematic IgM against KLH in the serum of γδ T cell-depleted group decreased significantly compared with that of KLH-immunized control group. This finding was similar to αβ T cell-depleted group that served as a positive control treated with anti-α and anti-β Abs. However, the production of IgM in non-related IgG-treated control groups did not decrease significantly (Figure 7C). These results confirmed the function of zebrafish γδ T cells in the initial CD4^+^ T cell activation, subsequent B cell proliferation, and Ab production during the complete activation of adaptive immunity. An adoptive transfer assay was performed to further validate this finding. The generated sibling recipient fish were administered with anti-MHC-II antibody thrice to eliminate the basal MHC-II^+^ APCs *in vivo*. With this method, the role of γδ T cells can be evaluated. The amount of MHC^+^ APCs in the depletion group was reduced by approximately 57% compared with that in the PBS- or non-related rabbit IgG-treated control groups (Figure 8A). The double elimination of MHC-II^+^ APCs and αβ T cells (reduced ~60%, Figure 8A) was also conducted by co-administering anti-MHC-II with anti-α, and anti-β Abs in the recipient fish. The sorted primary γδ T cells were then loaded *in vitro* with KLH and transferred into the KLH-stimulated sibling recipient fish. The decreased production of antigen-specific IgM secondary to the depletion of MHC-II^+^ APCs was optimally recovered by transferring KLH-activated γδ T cells in a dose-dependent manner. By contrast, no significant improvement was observed through the transfer of inactivated γδ T cells (Figures 8B,C). Moreover, the decrease in IgM *via* the double depletion of MHC-II^+^ APCs plus αβ T cells was no longer restored by transferring either activated or inactivated γδ T cells. Therefore, γδ T cells contributed to antigen-specific IgM production in αβ T cell-dependent pathways.

### γδ T Cells Involve in Mucosal IgZ Production

**Figure 9:**
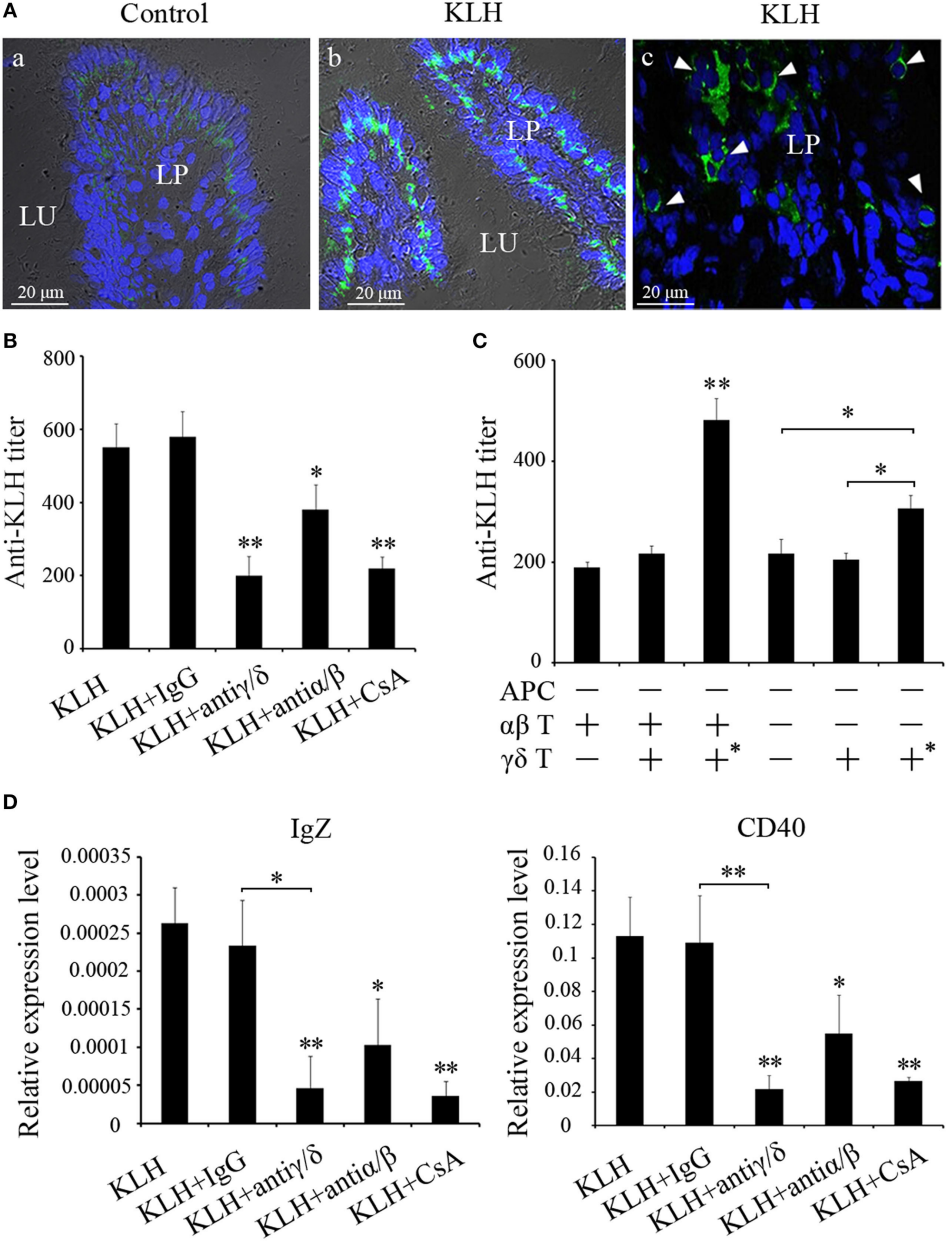
**Evaluation of zebrafish γδ T cells in mucosal IgZ production**. **(A)** Differential interference contrast images of immunofluorescence staining of zebrafish hind-gut cryosections from control fish (a) and fish that had been stimulated with KLH (b), stained for *Dr*TRGC (green) and nuclei are stained with DAPI. Enlarged images of the LP (c), without differential interference contrast, showing infiltrating γδ T cells that marked with white arrows in the gut lamina propria. LP represents lamina propria and Lu represents gut lumen. **(B)** The titers of IgZ against KLH in each treatment group were examined by ELISA. The control groups immunized with KLH only. **(C)** ELISA for KLH-specific IgZ Abs in the intestine mucus from the recipient fishes with adoptive transfer in different-treatment combinations. All the treatments and combinations were consistent to that shown in Figure 8B. The titer was ascertained based on the highest serum dilution at which the A450 ratio (A450 of postimmunization sera/A450 of preimmunization sera) is ≥ 2.1. The data of the background control group with PBS administration were subtracted from each experimental group. **(D)** The expression levels of IgZ and CD40 in the metenteron were determined by real-time PCR. Means ± SD of three independent experiments are shown. *P < 0.05, **P < 0.01.

The abundance of γδ T cells in mucosal tissue is reminiscent of some connection between γδ T cells and mucosal immunity. To test this hypothesis, we initially observed the distribution of γδ T cells in the posterior intestine, which have been considered to play an important role in the mucosal immune responses of fish. Immunofluorescence demonstrated that γδ T cells were prevalent in the gut, and the γδ T cells considerably accumulated in the lamina propria of the stimulated fish (Figure 9A). The antigen-specific IgZ response was subsequently examined in the γδ T cell-depleted group. Compared with the KLH-immunized control group, the KLH-immunized group administered with non-related IgG (negative control) produced almost identical IgZ levels. Conversely, the levels of IgZ antibody were lower in the γδ T cell-depleted group, the CsA addition group, and the αβ T cell-depleted group (Figure 9B). The mRNA expression levels of IgZ were similarly downregulated in the hind-gut of γδ T cell-depleted and CsA-administered groups. The alteration of IgZ in various experimental groups was accompanied by a similar change in CD40 expression. This finding implied that CD40-mediated co-stimulatory regulation may be involved in the IgZ production (Figure 9D). In the adoptive experiment, antigen (KLH)-specific IgZ production can be restored to a normal level when KLH-pulsed γδ T cells are transferred in the presence of αβ T cells. KLH-specific IgZ production can also recover to a certain degree in the absence of αβ T cells in the αβ T cell-depleted group (Figure 9C). These results imply that αβ T cell-dependent mechanism may be largely responsible for antigen-specific mucosal IgZ production; however, there might also exist an αβ T cell-independent mechanism underlying IgZ production.

## Discussion

Since the discovery of γδ T cells in the mid-1980s in humans, these cells have been considered as an enigmatic population that exhibits the characteristics of both innate and adaptive immune functions, such as participating in rapid resistance to pathogen infections, acting as an APC to initiate adaptive immunity, and performing CD4^+^ Th and CD8^+^ CTL-like functions (4, 30). Thus, the γδ T cells are thought to be a more primordial lymphocyte subset with a long evolutionary history throughout vertebrate evolution (31). Ancient vertebrates should also be included to obtain additional insights regarding vertebrate evolution. Teleost fishes possess the recognizable adaptive immunity on the basis of their complicated innate immune system; as such, they are potential organisms to investigate the evolutionary history of vertebrate immune systems (32). In our study, the γδ T cells from a zebrafish model were described through molecular and functional characterization. TCR-γ and TCR-δ molecules were initially identified from zebrafish to show various conserved structural features of their mammalian counterparts, as determined by similar genome synteny, gene organization, and existence of the hallmarks of typical TCR sequences. Zebrafish γδ T cells exhibited the surface phenotype of γ^+^ δ^+^CD4^−^CD8^+^, which is similar to that of mammalian γδ T cells, by immunofluorescence staining. The magnetically sorted zebrafish γδ T cells manifested lymphocyte behaviors, as shown by their distribution in the lymphoid gate in FCM analysis, and their expression of the hallmark molecules exclusive from that of αβ T cells, mIgM^+^ B cells, and monocytes/macrophages through RT-PCR. Tissue distribution analysis demonstrated that the zebrafish γδ T cells yielded the highest localization in the skin, gills, and intestine, three mucosal-associated tissues, which coincides with the supposed function of γδ T cells prominent in mucosal immunity. Zebrafish γδ cells possess typical lymphocyte morphology, with round dense nuclei surrounded by a thin round ring of cytoplasm, a similar diameter, and numerous surface frills, as determined by TEM and SEM. Functionally, zebrafish γδ T cells manifested potent non-specific phagocytic abilities for both soluble and particulate antigens and functioned as an APC to initiate the full activation of adaptive immunity, including priming antigen-specific CD4^+^ T cell activation, subsequent B cell proliferation, and systematic IgM production. Through *in vivo* depletion and transfer assays, the APC function of zebrafish γδ T cells was found to be in an αβ T cell-dependent pathway. These experimental lines suggested the presence of γδ T cells in zebrafish, which possess multi-faceted conservation of their mammalian γδ T cells.

As one of the most important cellular components in the immune system, the origin and evolution of the T and B lymphocytes have received considerable attention; however, this topic has long been challenged in immunology because of limited information regarding T and B lymphocytes in phylogenetically ancient species (33, 34). B cells in mammals were thought to originate from primitive phagocytic cells, such as macrophages in ancient vertebrates. This hypothesis was recently supported by the observations that B cells in teleost fish exhibit strong phagocytic and microbicidal abilities and act as a pivotal initiating APC in priming naive CD4^+^ T cells and subsequent adaptive humoral immunity (21, 26). These characteristics are typical myeloid traits and innate-like functional features shared by macrophages. The B-1 subset and the ancient B cell lineage have been suggested to have originated from a group of ancient phagocytic ancestors similar to macrophages because B-1 cells in mammals possess similar features to those of teleost B cells (35, 36). Moreover, two alternative pathways have been proposed to explain the evolutionary relationship between B-1 and B-2 cell lineages (34). However, the evolutionary history of the T lymphocytes should be clarified. In our study, zebrafish γδ T cells also possessed potent phagocytosis and APC function on the initiation of adaptive immunity; the performance was similar to that of B cells in teleost fish. These myeloid traits and innate features shared by zebrafish γδ T cells were also observed in a subtype of human Vγ9Vδ2 T cells (27, 37), which resembling the correspondence of teleost B cells to human B-1 lineage. Thus, teleost γδ T cells and human Vγ9Vδ2 T-like subset, as well as γδ T cells and B-1 lineage cells, possibly showed a close evolutionary relationship. Several phylogenetic studies favored the γδ TCR having evolved before both the αβ TCR and B cell Igs because of the following reasons (31): first, the evolutionary trees based on the C region sequences of four TCRs and two Igs suggest that TCR evolved earliest; then, they produce IgL and IgH; second, the primordial immune cells should be capable of recognizing a wide array of antigens. Third, tree topologies indicated that direct antigen recognition is more primitive than that of indirect antigen recognition, as αβ TCRs do. Membrane-bound forms were regarded as an ancestral characteristic, and the secretion of Igs was likely a derived trait. These findings point the finger at the origin of T and B lymphocytes, which ultimately attribute to the origin of TCR and BCR. Taken together, it seems reasonable to assume that T cells were probably ancestral immune cells; γδ T-like cells may be a more primordial subpopulation that gave rise to αβ T cells and B cells. In addition, γδ T and B cells might have originated from a common phagocytic progenitor. The exact origins of γδ T cells, αβ T cells, and B cells can be fully elucidated when these cells are subjected to further cellular and functional identifications in other ectothermic vertebrates.

Typical adaptive humoral immunity employed in higher vertebrates was initially established in teleost fish on the basis of the origin of mIgM^+^ B cells, CD4^+^ Th2-like cells, and the oldest Ig of IgM (8). However, the cellular regulatory mechanisms underlying teleost adaptive humoral immunity remain limited. A dendritic cell-like and a B-1 cell-like population have been functionally identified from several fish species, and these cells could act as APCs for antigen-specific T cell activation and IgM production (10, 21, 38). In this study, γδ T cells in zebrafish could promote antigen-specific CD4^+^ T cell activation and IgM production; thus, a previously unknown cellular member was added to the APC family of teleost fish. IgM is a primitive antibody from teleost fish, and a similar function was characterized in a subtype (Vγ9Vδ2 T) of human γδ T cells (37, 39). Therefore, γδ T cells in IgM production may be initially participate in adaptive humoral immunity, and this function was conserved from fish to mammals throughout vertebrate evolution. Along with other Ig isotypes, such as IgA, IgE, IgD, and IgG, γδ T cells and their function may expand correspondingly, as evidenced by the observations that mice deficient in individual γδ T subsets can manifest changes in serum Abs, including major subclasses (40). γδ T cells also play essential roles in IgZ (also named IgT) production, a novel Ig recently identified from zebrafish, rainbow trout, and several other fish species such as common carp (28, 41, 42). This IgZ/T molecule has been observed as the prevalent Ig in all fish mucosa-associated lymphoid tissues (MALTs) examined, including gut-associated lymphoid tissue, skin-associated lymphoid tissue, gill-associated lymphoid tissue, and nasopharynx-associated lymphoid tissue in rainbow trout, the latter of which is a newly discovered MALT in teleost carrying conserved anatomical structures and defense functions that serve as an important mucosal immune barrier against pathogens in the olfactory system across vertebrate species (28, 43–47). Thus, teleost fish is a potential model system for the exploration of precise cellular and molecular mechanisms underlying mucosal immunity because fish have unique and highly developed mucosa immune systems for the defense against numerous microorganisms in aquatic environment. Using zebrafish model, the functions of γδ T cells in mucosal immune responses could be greatly improved.

In mammals, γδ T cells have found to be a group of highly heterogeneous cells with a variety of subtypes. They are abundant in the epithelia of various tissues such as skin, small intestine, and reproductive tract. However, γδ TCR usage is usually not equivalent in different epithelia. For example, the predominant receptor of dermal epithelia using Vγ5 is not used in the gut epithelium, and the predominantly used γ gene in intestinal intraepithelial lymphocytes (I-IELs) is Vγ7, the functional use of which has not been conspicuous elsewhere (48). Unlike the other epithelial γδ cells, the I-IELs can employ a variety Vδ segments, with Vδ4 being the most prevalent, and Vδ5, Vδ6, and Vδ7 being used less frequently (49–51). Besides, the junctions of both the γ and δ chains exhibit considerable junctional diversity, including extensive N-regions and the use of both D elements in many of the δ chains (49, 50). It makes I-IELs have a very large potential antigenic repertoire. So far, studies on fish γδ TCR repertoire and its functional diversity are very limited. Further investigations are needed to address whether the differentiation of VγVδ TCR chains, and γδ T subtypes had been existed in teleost fish. Clarification on these issues would be benefit to provide valuable insights into the functional evolution of γδ T cell subset.

In conclusion, our studies provided the first evidence confirming that γδ T cells had existed in teleost fish. These cells play an essential role in the initiation of adaptive immunity. Our study also provided insights into the origin of T cell subset in ancient vertebrates and the functional conservation of γδ T cells during evolution. Given γδ T cells play pivotal roles in various immune-relevant diseases, such as rheumatoid arthritis, inflammatory bowel disease, systemic lupus erythematosus, multiple sclerosis, diabetes, and autoimmune thyroid and liver disorders (52). Zebrafish is a potential model organism that can be used to investigate the biological characteristics of γδ T cells, γδ T-mediated diseases, and clinical therapies.

## Author Contributions

Conceive and designed the experiments: J-zS and FW. Performed the experiments: FW, C-bH, and KG. Analyzed the data: FW and J-xM. Contributed reagents/materials/analysis tools: L-xX and J-zS. FW wrote the manuscript.

## Conflict of Interest Statement

The authors declare that the research was conducted in the absence of any commercial or financial relationships that could be construed as a potential conflict of interest.

## Abbreviations

*Dr*TRGC, *Danio rerio* T cell receptor gamma constant region; *Dr*TRDC, *Danio rerio* T cell receptor delta constant region; *Dr*TRAC, *Danio rerio* T cell receptor alpha constant region; *Dr*TRBC, *Danio rerio* T cell receptor beta constant region; KLH, keyhole limpet hemocyanin; MHC-II, MHC class II; CD4^+^ T_KLH_, KLH-stimulated CD4^+^ T cell; BLAST, basic local alignment search tool; FITC-KLH, FITC-conjugated KLH; FITC-A.h, FITC-labeled *Aeromonas hydrophila*; FCM, flow cytometry; CsA, cyclosporin A; *Dr*CD4, *Danio rerio* CD4; *Dr*CD80/86, *Danio rerio* CD80/86; *Dr*CD83, *Danio rerio* CD83; *Dr*MHC-II, *Danio rerio* MHC class II; *Dr*IgM, *Danio rerio* IgM; *Dr*CD40, *Danio rerio* CD40; *Dr*CD154, *Danio rerio* CD154; *Dr*IgZ, *Danio rerio* IgZ.
