# Supplementary material for: Characterization of γδ T Cells from Zebrafish Provides Insights into Their Important Role in Adaptive Humoral Immunity
Source: Front Immunol. 2017 Jan 9;7:675. doi: 10.3389/fimmu.2016.00675 (PMC5220103; doi:10.3389/fimmu.2016.00675)
Supplement: Supplementary file 1 [file Data_Sheet_1.PDF]

Supplementary Information for

Characterization of γδ T cells from zebrafish provides insights into their important role in adaptive humoral immunity

Feng Wan[1], Chong-bin Hu[1], Jun-xia Ma[1], Ke Gao[1], Li-xin Xiang[1], and Jian-zhong Shao[1,2]

[1]College of Life Sciences, Zhejiang University, Hangzhou 310058, People's Republic of China; Key Laboratory for Cell and Gene Engineering of Zhejiang Province, Hangzhou 310058, People's Republic of China;

[2]Laboratory for Marine Biology and Biotechnology, Qingdao National Laboratory for Marine Science and Technology, People's Republic of China

Corresponding Author: Jian-zhong Shao and Li-xin Xiang, Zhejiang University, Hangzhou 310058, Zhejiang, People's Republic of China. Telephone/Fax: +86-571-88206582. E-mail address: shaojz@zju.edu.cn; xianglx@zju.edu.cn

Supplementary Table1. Primers used for gene cloning and expression analyses

Primer Name Accession Number/Ref	Sequence (5' to 3')	Application
*Dr*TRAC-F1 AF425590.1	AAACTGAAGTGAAGCCGAATA	ORF cloning
*Dr*TRAC-R1 AF425590.1	AGACGTTAGCTCATCCACGCTTT	ORF cloning
*Dr*TRBC-F1[(16)]	AATTCCCAAGAGTGAACCATCCG	ORF cloning
*Dr*TRBC-R1[(16)]	TCATTTGAACCCTTTCGCCATAC	ORF cloning
*Dr*TRGC-F1 AY973884.1	TAGTAACTGAACCTGGGAAGGAC	ORF cloning
*Dr*TRGC-R1 AY973884.1	TTAAGAGCGCTCCTCTTTCTTTCC	ORF cloning
*Dr*TRDC-F1 CAK05280.1	AAGAGACAGTGAATTCTCCTCC	ORF cloning
*Dr*TRDC-R1 CAK05280.1	ATTACAAAGATCAGAGCCTCAAT	ORF cloning
*Dr*TRGC-F2 KX009744	CGGGATCCTAGTAACTGAACCTGGGAAGGAC	In situ hybridization
*Dr*TRGC-R2 KX009744	CCCTCGAGTTAAGAGCGCTCCTCTTTCTTTCC	In situ hybridization
*Dr*TRDC-F2 KX009743	CGGGATCCAAGAGACAGTGAATTCTCCTCC	In situ hybridization
*Dr*TRDC-R2 KX009743	CCCTCGAGAAACAAAAATGCCTTTATAGAC	In situ hybridization
*Dr*TRGC-F3 KX009744	TAGTAACTGAACCTGGGAAGGAC	Real-time RT-PCR
*Dr*TRGC-R3 KX009744	TTAAGAGCGCTCCTCTTTCTTTCC	Real-time RT-PCR
*Dr*TRDC-F3 KX009743	GTGGCCGCCGGATTCTTTCCTCA	Real-time RT-PCR
*Dr*TRDC-R3 KX009743	TTTGTGGATGGTGGGGTGGTAGT	Real-time RT-PCR
*Dr*TRAC-F2 AF425590.1	CCGGAATTCAAAACTGAAGTGAAGCCGAATA	Prokaryotic expression
*Dr*TRAC-R2 AF425590.1	CCGCTCGAGGTTGACTTTTTCATCTTTTTCTAAATC	Prokaryotic expression
*Dr*TRBC-F2[(16)]	CCGGAATTCGATCCTTTAATTCCCAAGAGTGAAC	Prokaryotic expression
*Dr*TRBC-R2[(16)]	CCGCTCGAGCTTCATCCACTGTGACGACTTCAC	Prokaryotic expression
*Dr*TRGC-F4 KX009744	CCGGAATTCGAACCTGGGAAGGACAGTGTTGTG	Prokaryotic expression
*Dr*TRGC-R4 KX009744	CCGCTCGAGAAACAGCTGCAGACTCTGTTTCTTCTC	Prokaryotic expression
*Dr*TRDC-F4 KX009743	CCGGAATTCTTGTCTGTCTTGTCCCCTATAAAG	Prokaryotic expression
*Dr*TRDC-R4 KX009743	CCGCTCGAGGTTTGTTTTTGGATCATCATTATTTG	Prokaryotic expression
*Dr*MHC-II-F L19446.1	CTCTCATTGAACTATGGATC	Gene expression
*Dr*MHC-II-R L19446.1	CAAAGCTGTCAGTTGCAGTTG	Gene expression

*Dr*CSF-1R-F NM_131672	GACCTGCTGAACTTCCTGCG	Gene expression
*Dr*CSF-1R-R NM_131672	GACTACGTAGTTGGAGTCGTTC	Gene expression
*Dr*FcεRIγ-F EF158447.1	TCTGGATGGGATCCTGATCGTTTAC	Gene expression
*Dr*FcεRIγ-R EF158447.1	CATGGTTACAGCAAAATTGATAG	Gene expression
*Dr*CD4-F EF601917.1	GCCCCTTCTCCAGCAGAT	Gene expression
*Dr*CD4-R EF601917.1	GATGGCAACACAAAGC	Gene expression
*Dr*CD8α-F BC162235.1	AAAAGGACAGACAGCGGACTA	Gene expression
*Dr*CD8α-R BC162235.1	GTTGCGATCAGCAGAACGAG	Gene expression
*Dr*IgM-F1 AF281479.1	TGAGCACAATAAGCGGAAAG	Gene expression
*Dr*IgM-R1 AF281479.1	GCCAAGTCACAAACACCT	Gene expression
*Dr*β-actin-F AF057040.1	AGGTCATCACCATCGGCAAT	Gene expression
*Dr*β-actin-R AF057040.1	GATGTCCACGTCGCACTTCA	Gene expression
*Dr*IgM-F2 AF281479.1	TGAGCACAATAAGCGGAAAG	Real-time RT-PCR
*Dr*IgM-R2 AF281479.1	GCCAAGTCACAAACACCT	Real-time RT-PCR
*Dr*CD40-F FJ172754	TTCTCAGAGTCTGTTTGCTGGTCAC	Real-time RT-PCR
*Dr*CD40-R FJ172754	TCCCGTCACTACATTCCTCAC	Real-time RT-PCR
*Dr*IgZ-F AY643752.1	AAAAGTCACCTGTACCACCCG	Real-time RT-PCR
*Dr*IgZ-F AY643752.1	GATCACTGGATTTGCCCTCT	Real-time RT-PCR
*Dr*CD154-F FJ534590	CGAATGGCAACAGGGCACAAGAATG	Real-time RT-PCR
*Dr*CD154-R FJ534590	TCTAAACACTCCTGCTGATGATGCC	Real-time RT-PCR
*Dr*LcK-F AY390224	AGATGAATGGTGTGACCAGTGTA	Real-time RT-PCR
*Dr*LcK-R AY390224	GATCCTGTAGTGCTTGATGATGT	Real-time RT-PCR

F, forward primer; R, reverse primer.

Supplementary Table 2. The antigenic epitopes of the genes

Name	Sequence
*Dr*TRAC	TEVKPNIYKVGNSC
*Dr*TRBC	EGKKYTISSRLKVSQKMWKKSTN
*Dr*TRGC	STETPISQESANPEKKQ
*Dr*TRDC	SVLSPIKGHGSDIC
CD8α	IQGVPDPVTQGP

Supplementary FIGURE 1

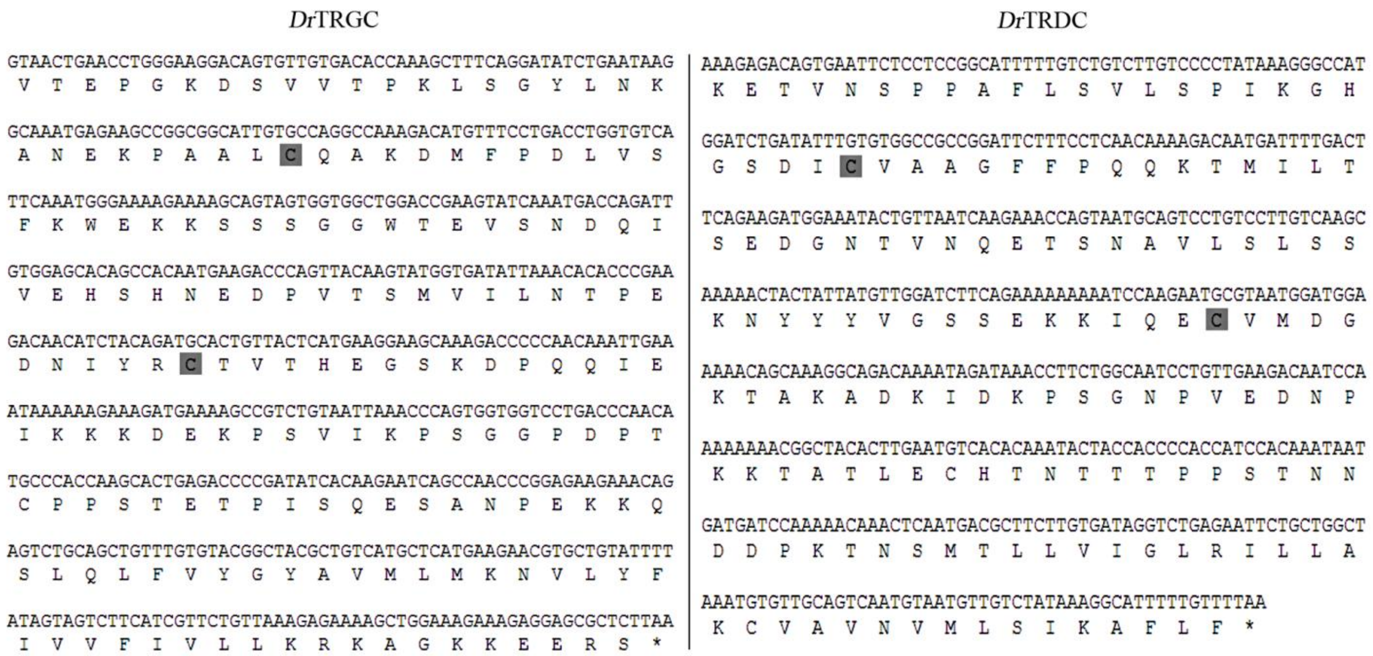

Supplementary FIGURE 1. cDNA sequences and deduced amino acids of *Dr*TRGC and *Dr*TRDC. The cDNA of *Dr*TRGC consists of 540 bp comprising a 537 bp ORF that encodes 179 amino acids, and the *Dr*TRDC cDNA consists of 471 bp comprising a 468 bp ORF that encodes 156 amino acids. The *Dr*TRGC and *Dr*TRDC proteins are predicted to have molecular weights of ~20 KDa and ~17 kDa, respectively. Two conserved cysteines in the Ig-C domain of *Dr*TRGC and *Dr*TRDC, which are involved in an intra-chain disulfide bond, are shown in dark gray. The asterisk

represents the stop codon.

Supplementary FIGURE 2

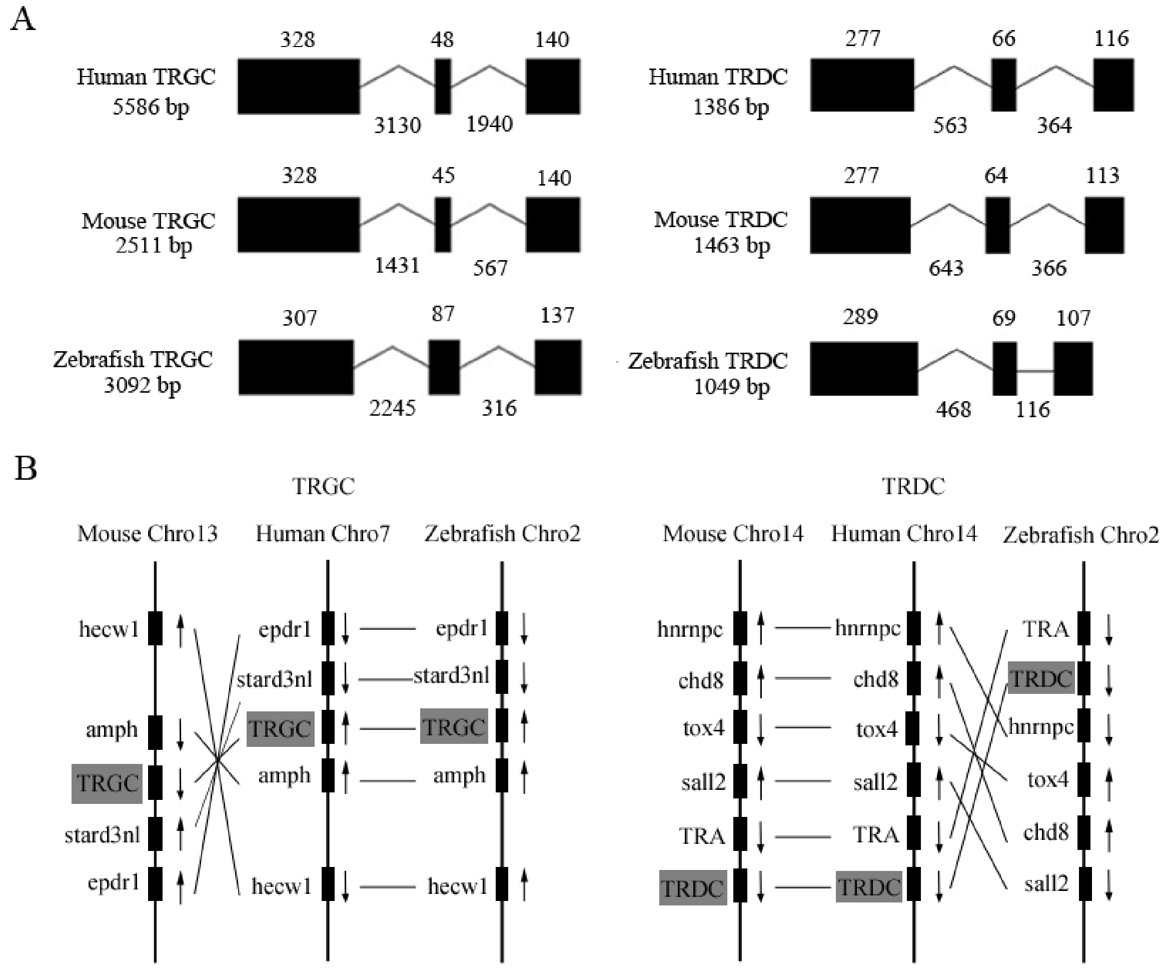

Supplementary FIGURE 2. Comparative analyses of the chromosomal locations and organizations of *Dr*TRGC and *Dr*TRDC genes in human, mouse, and zebrafish. (A) Comparison of the exon/intron organizations of the *Dr*TRGC and *Dr*TRDC genes in human, mouse, and zebrafish. The rectangles represent the exons, whereas the lines between them indicate the introns. The sizes of the exons and introns are shown by the numbers above and below, respectively; and the sizes of the whole *Dr*TRGC and *Dr*TRDC are shown under the gene name. (B) Genes are linked with lines and aligned in columns to facilitate visualization of synteny. The arrows on the right side of the

gene names indicate the gene orientation.

Supplementary FIGURE 3

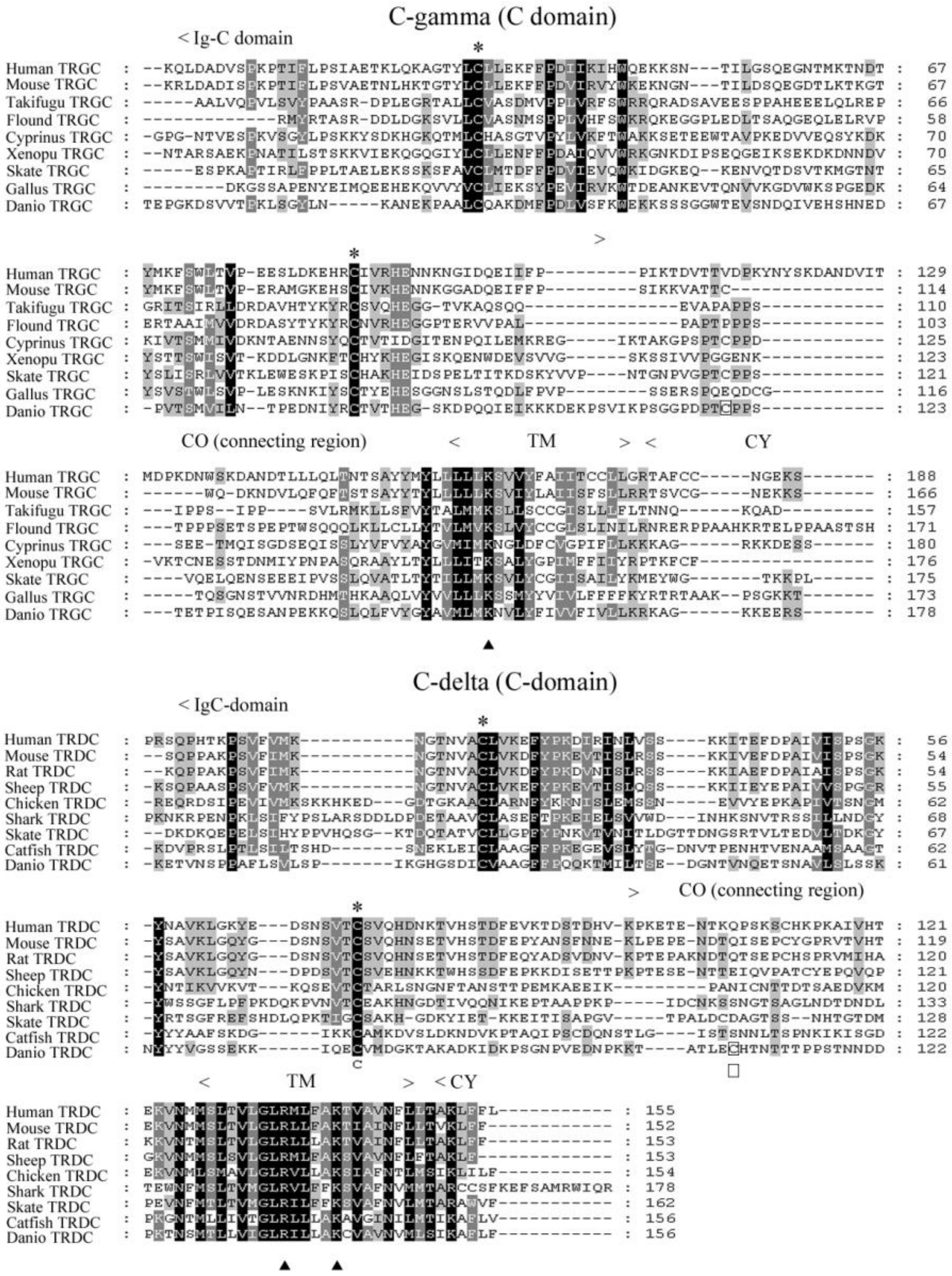

Supplementary FIGURE 3. Alignment of the predicted amino acid sequences of the *Dr*TRGC and *Dr*TRDC with those of corresponding proteins of other species. Residues shaded in black are completely conserved across all species, and residues shaded in gray are similar with respect to the side chains. The dashes in the amino acid sequences indicate gaps introduced to maximize alignment. The predicted Ig-C domain, connecting region (CO), transmembrane domain (TM), and cytoplasmic domain (CY) boundaries are indicated above. Conserved cysteine residues that form the intra-chain disulfide bonds are marked with asterisks, and the cysteines that form the inter-chain disulfide bonds are boxed. Black triangles indicate conservative arginine and lysine in the TM. Gene accession numbers are noted in the legend of Supplementary Information FIGURE 4.

Supplementary FIGURE 4

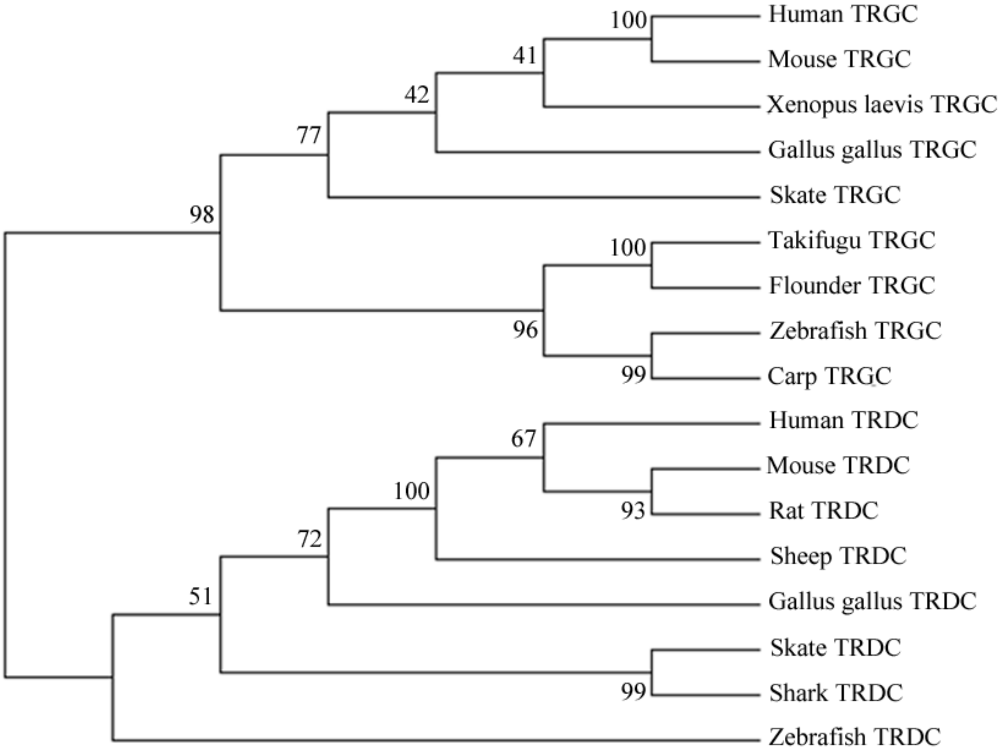

Supplementary FIGURE 4. An unrooted phylogenetic tree of protein sequence of was constructed by the neighbor-joining method using MEGA 5.0. Node values

represent percentage bootstrap confidence derived from 1000 replicates. GenBank accession numbers are as follows: Human TRGC, S01895, (http://www.ncbi.nlm.nih.gov/protein/S01895); Mouse TRGC, AAA51271, (http://www.ncbi.nlm.nih.gov/protein/AAA51271); Xenopus laevis TRGC, AF440821, (http://www.ncbi.nlm.nih.gov/protein/20385990); Gallus gallus TRGC, AAA87009, (http://www.ncbi.nlm.nih.gov/protein/AAA87009); Skate TRGC, AAB51498, (http://www.ncbi.nlm.nih.gov/protein/AAB51498); Takifugu TRGC, BAE16941, (http://www.ncbi.nlm.nih.gov/protein/BAE16941); Flounder TRGC, BAC65460, (http://www.ncbi.nlm.nih.gov/protein/BAC65460); Carp TRGC, ABD04398, (http://www.ncbi.nlm.nih.gov/protein/ABD04398); Zebrafish TRGC, KX009744; Human TRDC, A35591, (http://www.ncbi.nlm.nih.gov/protein/A35591); Mouse TRDC, AAA51274, (http://www.ncbi.nlm.nih.gov/protein/AAA51274); Rat TRDC, CAB58999, (http://www.ncbi.nlm.nih.gov/protein/6093263); Sheep TRDC, CAC14678, (http://www.ncbi.nlm.nih.gov/protein/11071829); Gallus gallus TRDC, AF175433_1, (http://www.ncbi.nlm.nih.gov/protein/5802623); SharkTRDC, AAA87016, (http://www.ncbi.nlm.nih.gov/protein/974232); Skate TRDC, AAB51497,(http://www.ncbi.nlm.nih.gov/protein/1929970); Zebrafish TRDC, KX009743.

Supplementary FIGURE 5

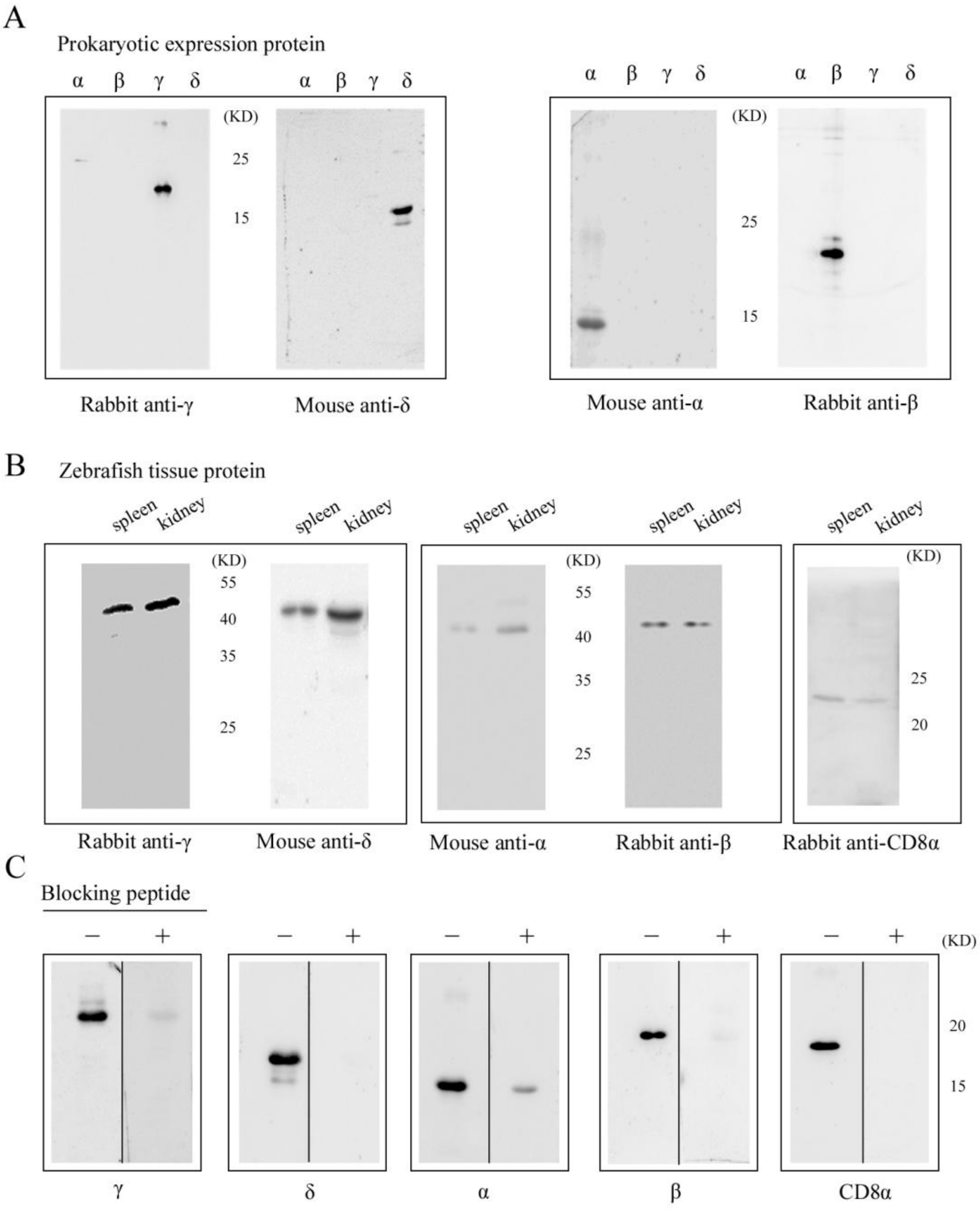

Supplementary FIGURE 5. Western blot detect the specificity and cross-reactions of the purified Abs. (A) Western blot show the anti-γ, -δ, -α and -β can bind to the corresponding recombinant proteins (the whole bacterial protein) and have no cross-reaction with other TCRs. The recombinant proteins of *Dr*TRGC, *Dr*TRDC, *Dr*TRAC and *Dr*TRBC are predicted to be ~21 KDa, ~15 KDa, ~14 kDa and ~19 kDa,

respectively. The position of MW marker is shown on the side of the PVDF membranes. (B) Western blots show the anti-γ, -δ, -α, -β and -CD8α can specifically bind to the corresponding endogenous target proteins in spleen and kidneys respectively. (C) Western blots detect the effects of blocking peptides on all the produced Abs. The recombinant proteins were incubated either with anti-γ, -δ, -α, -β, and -CD8 Abs respectively; or with these Abs plus their respective blocking peptides (5 μg/mL). The molecular weights of *Dr*TRGC, *Dr*TRDC, *Dr*TRAC, *Dr*TRBC, and CD8α are predicted to be ~21 KDa, ~15 KDa, ~14 kDa, ~19 kDa, and ~17 KDa, respectively.

Supplementary FIGURE 6

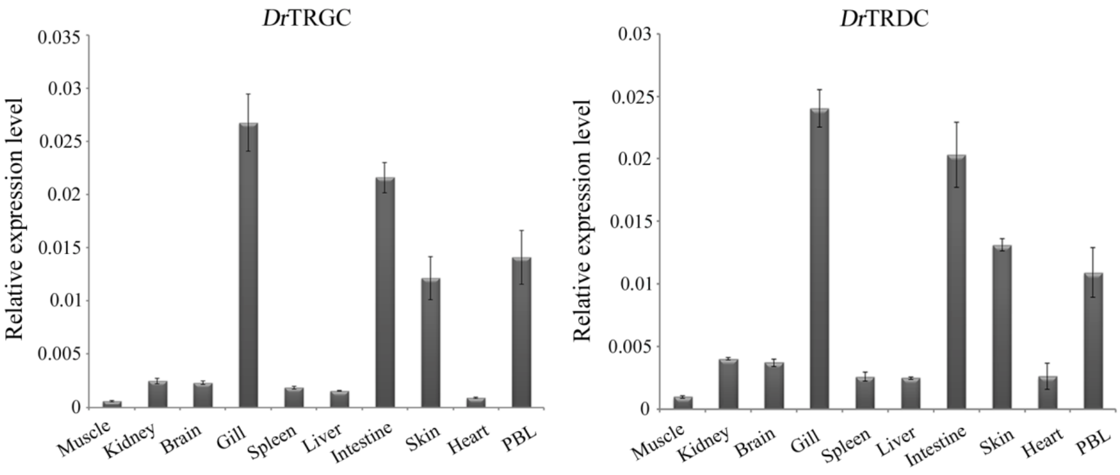

Supplementary FIGURE 6. Expression analysis of *Dr*TRGC and *Dr*TRDC transcripts in various adult tissues. Relative gene expression of *Dr*TRGC and *Dr*TRDC transcripts in various adult tissues (heart, spleen, liver, intestines, kidneys, gills, brain, skin, muscles, and PBL). The relative expression value was averaged from three duplicates, each of which contains 10–15 fish.

Supplementary FIGURE 7

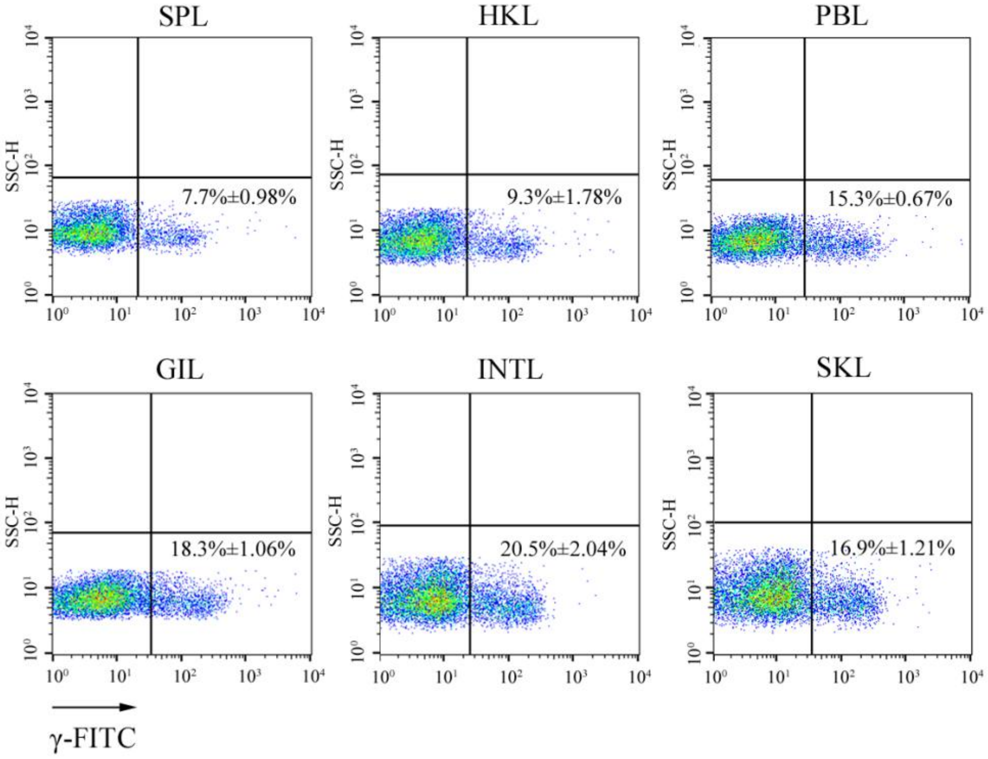

Supplementary FIGURE 7. Tissue distribution of γδ T cells in zebrafish was analyzed by FCM. Leucocytes were isolated from spleen (SPL), kidneys (HKL), peripheral blood (PBL), skin (SKL), gills (GIL), and intestines (INTL) by Ficoll-Hypaque centrifugation. Numbers in each panel indicate percentage of γ^+ cells (bottom right).
